# Supplementary material for: A gene with a thousand alleles: The hyper-variable effectors of plant-parasitic nematodes
Source: Cell Genom. 2024 May 29;4(6):100580. doi: 10.1016/j.xgen.2024.100580 (PMC11228951; doi:10.1016/j.xgen.2024.100580)
Supplement: Document S1. Figures S1‒S6 and Tables S1 and S2 [file mmc1.pdf]

**Cell Genomics, Volume *4***

## Supplemental information

# A gene with a thousand alleles:

# The hyper-variable effectors

# of plant-parasitic nematodes

**Unnati Sonawala, Helen Beasley, Peter Thorpe, Kyriakos Varypatakis, Beatrice Senatori, John T. Jones, Lida Derevnina, and Sebastian Eves-van den Akker**

## Supplemental Information

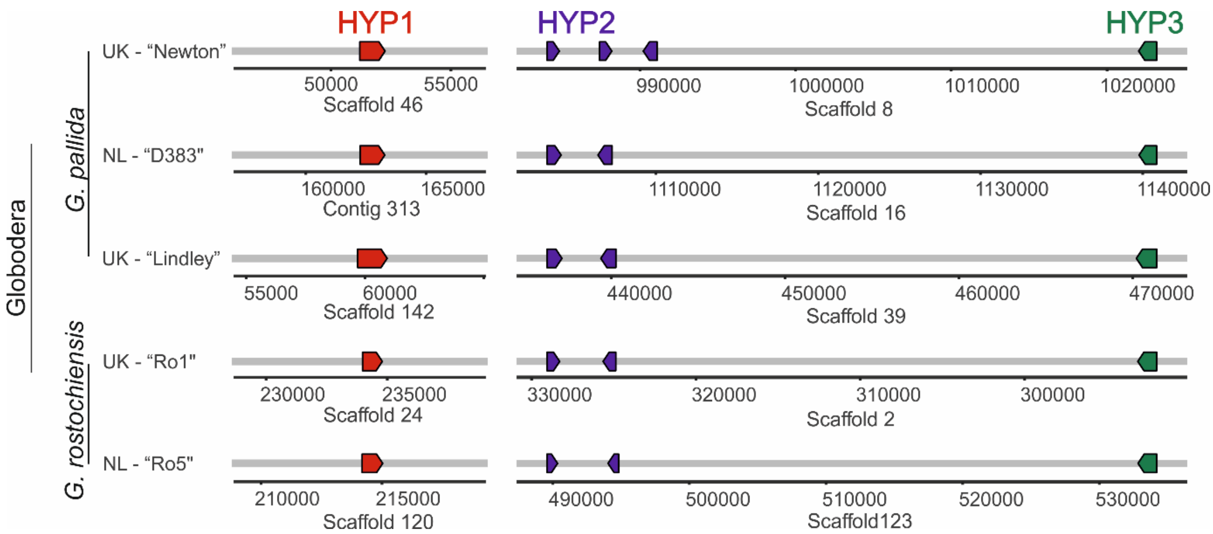


**Figure S1. Schematic gene maps indicating positions of HYP1, HYP2 and HYP3 loci across multiple populations/pathotypes of *G. pallida* and *G. rostochiensis,* related to Figure 1.**

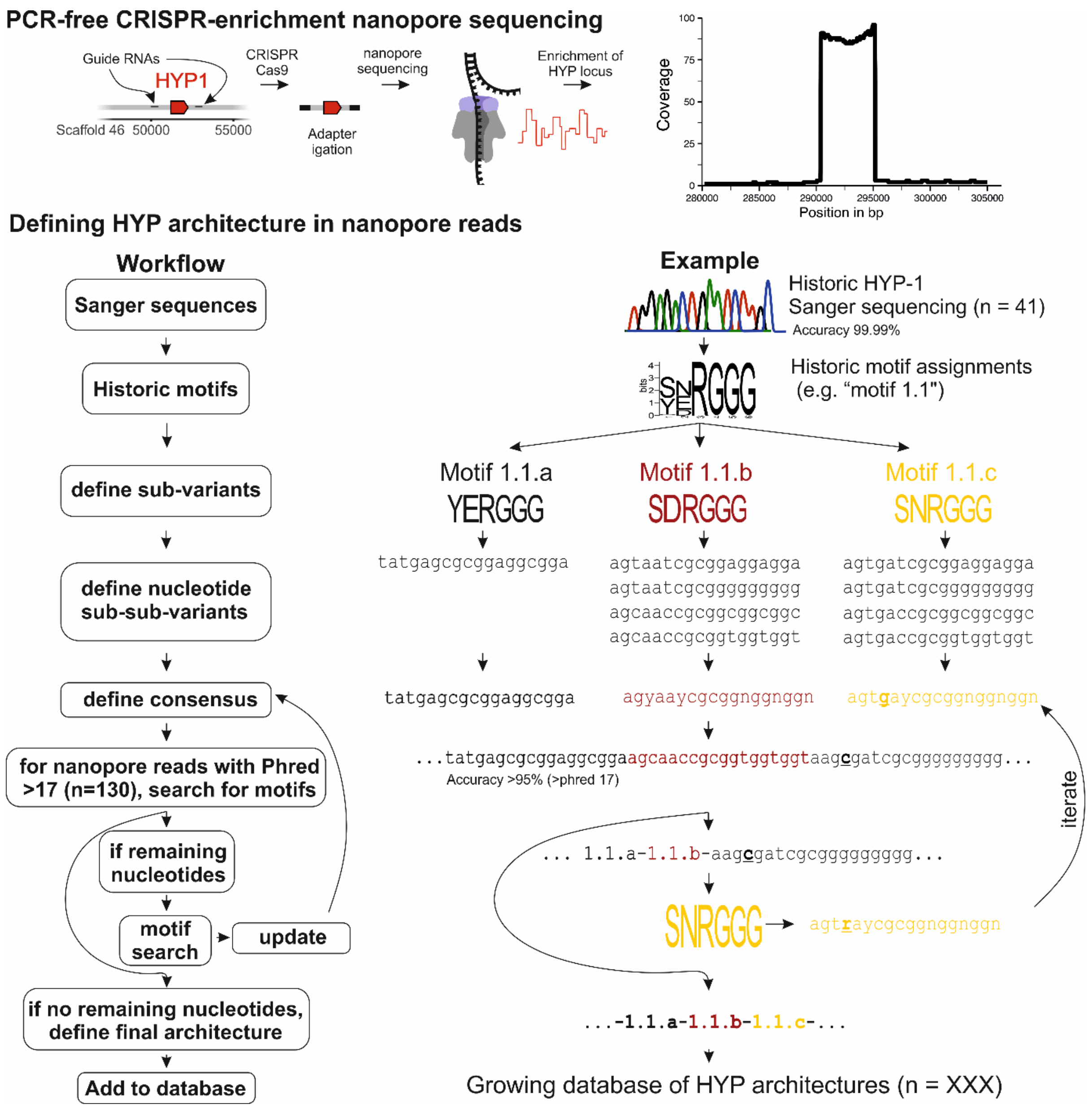


**Figure S2. Schematic of workflow for characterization of HYP variants within a population or single nematode, related to Figure 2.** A customised workflow was designed by taking advantage of the ‘modular’ nature of the motifs within the variable region of HYP genes. Previously identified motifs or those identified by MEME were used to build a pattern matching code using the Biostrings package in R. The pattern matching was optimised for error-prone nature of long reads. Several iterations were performed to update code for missed motifs. Finally, known motifs missed due to multiple sequencing errors were manually curated to generate a final list of HYP ‘variants’ consisting of an uninterrupted string of identified motifs.

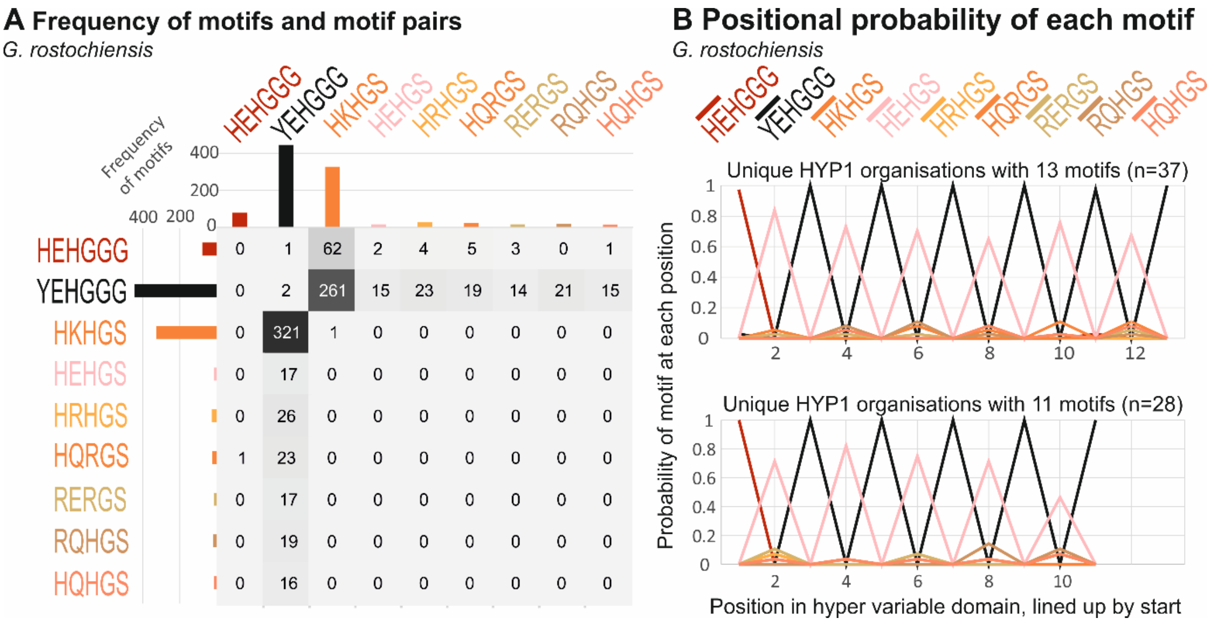


**Figure S3. There are different rules underlying *G. rostochiensis* HYP1 variation, related to Figure 2.** A) Frequency of motif pairs within HYP1 HVDs. Each motif is shown with amino acid sequence, y axis of matrix is position n and x axis is position n+1. For examples: i) HEHGGG is almost always first (and so almost never follows another motif), ii) all motifs can appear before YEHGGG, but YEHGGG is the only motif that can appear before RQHGS). B) The positional probability of each motif at each position for HYP1s with HVDs containing 13 (top) and 11 (bottom) motifs show an alternating pattern of YEHGGG followed by an other motif.

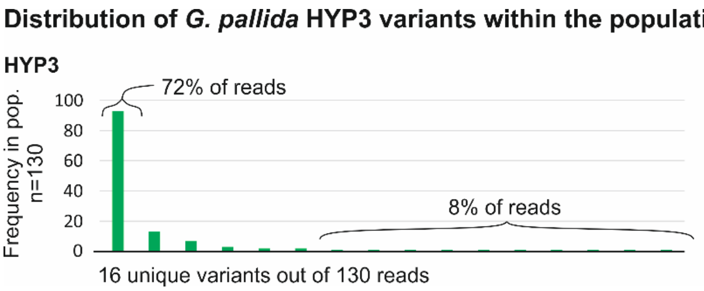


| | Method | |
|---|---|---|
| | Chao1 (± error) | ACE (± error) |
| Predicted no. alleles in pop. | 31 (13) | 48(4) |

**Figure S4. Frequency distribution and population size estimates of *G. pallida* HYP3 alleles, related to Figure 3.** The observed occurrences of each unique HYP variant when sampling a population (n= 125). Inset are two independent species abundance estimates for the total number of alleles in the population, based on the sampling.

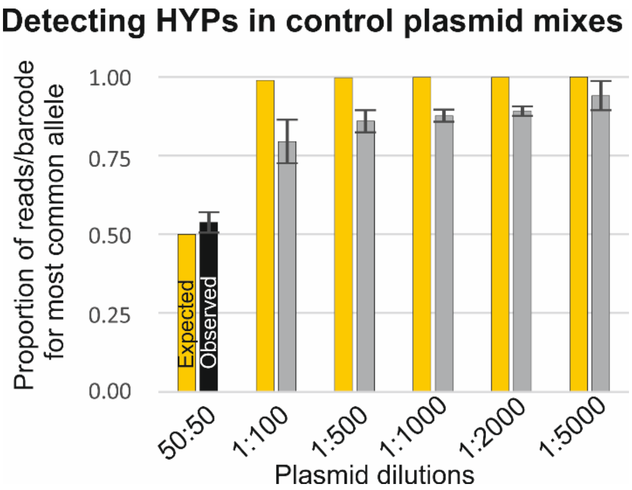


**Figure S5. Detecting HYPs in control plasmid mixes, related to Figure 4.** Comparison of observed (black and grey) and expected (yellow) proportions of reads per barcode pair that are contributed by the most common allele, for a range of known plasmid dilutions.

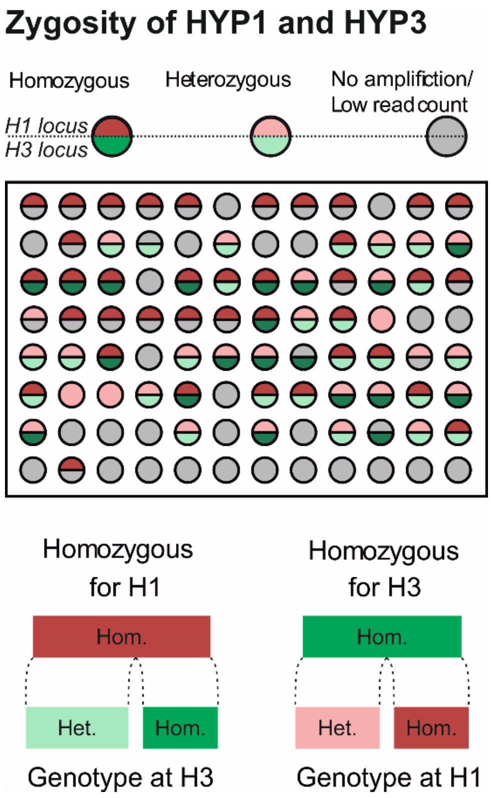


**Figure S6. Zygosity of HYP1 and HYP3 loci for 68 J2s, related to Figure 4.** Zygosity of HYP1 locus (reds) is unlinked to zygosity of HY3 locus (greens).

| Metrics | *G. pallida* 'Lindley'[5] shortread assembly | *G. pallida* 'Newton' longread assembly | *G. pallida* 'Lindley' longread assembly | *G. rostochiensis* 'Ro1'[2] shortread assembly | *G. rostochiensis* 'Ro1' longread assembly |
|---|---|---|---|---|---|
| **Size (Mb)** | 124.6 | 119.6 | 109.1 | 95.9 | 102.2 |
| **Scaffolds(n)** | 6,873 | 173 | 672 | 4281 | 267 |
| **Scaffold N50 (bp)** | 121,687 | 2,225,126 | 595,505 | 88,688 | 2,199,277 |
| **Longest scaffold (bp)** | 600,076 | 8,303,752 | 4,690,510 | 688,384 | 8,068,213 |
| **GC (%)** | 37 | 37 | 37 | 38 | 39 |
| **Ns (bp)** | 21,024,229 | 1,237,729 | 2,170,753 | 4,399,212 | 1,328,123 |
| **BUSCO (%)** | Complete:73% [Duplicated:13%], Fragmented:12%, Missing:14%, | Complete: 89% [Duplicated: 9.9%],Fragmented: 5.9%, Missing: 4.6% | Complete: 86% [Duplicated: 12%], Fragmented: 5.2%, Missing: 8.2% | Complete: 88% [Duplicated: 9.5%], Fragmented: 5.9%, Missing: 5.9% | Completed:87% [Duplicated:9.2%],Fragmented :5.9%,Missing:6.6% |
| **Predicted genes (n)** | 16,466 | 16,321 | 22,426 | 14,378 | 14309 |

**Table S1: Genome metrics for Globodera genome assemblies, related to Figure 1.**

**Table S2. Table of crRNAs used for targeting HYP1 and HYP3, related to Figure 1.**

| Target | Forward | Reverse |
|---|---|---|
| HYP1 (*G.pallida*) | ATTGTATGATTGTCAAAATCCGG | AATCAAATGACACATTACAATGG |
| HYP3 (*G.pallida*) | TTATGTCGATGCGTCCGAGTTGG | CGGATCCAACCCCCGGAACGCGG |
| HYP1 (*G.rostochiensis*) | AATAACTCCCGGCGACGTCGCGG | TCTCTAGTCCGAACGTCGAAAGG |
| HYP3 (*G.rostochiensis*) | CAGCCCAATTGCAGGATGTTTGG | CCGATTCTACGTCTTATGATAGG |
