# Supplementary material for: A gene with a thousand alleles: The hyper-variable effectors of plant-parasitic nematodes
Source: Cell Genom. 2024 May 29;4(6):100580. doi: 10.1016/j.xgen.2024.100580 (PMC11228951; doi:10.1016/j.xgen.2024.100580)
Supplement: Document S2. Article plus supplemental information [file mmc6.pdf]

Cell Genomics

Article

A gene with a thousand alleles: The hyper-variable effectors of plant-parasitic nematodes

Graphical abstract

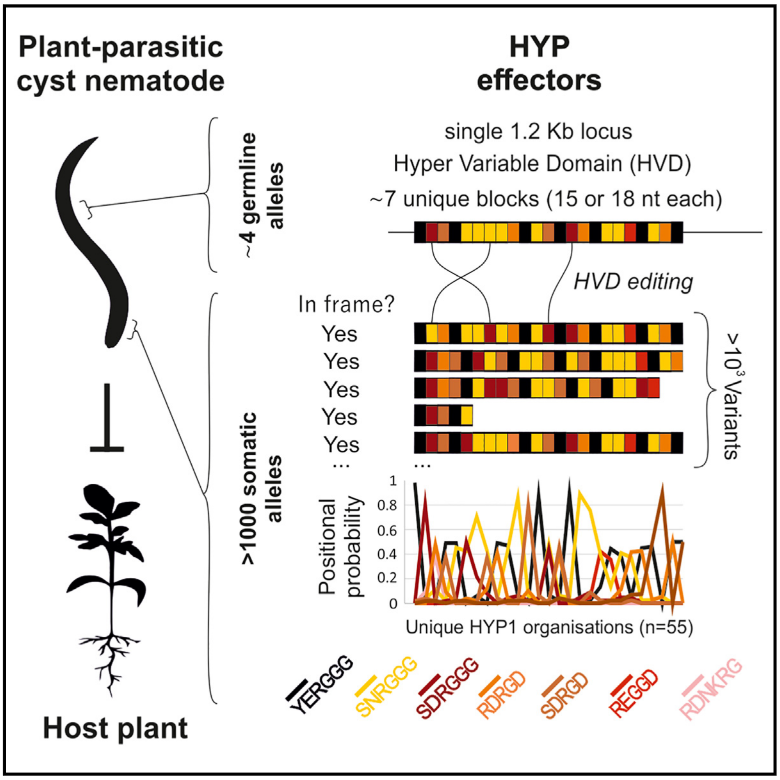

Highlights

- HYP effectors are necessary for parasitism in plant-parasitic potato cyst nematodes
- Single HYP genes consist of potentially thousands of alleles
- Alleles differ by apparent shuffling of motifs in the hyper-variable domain (HVD)
- We propose a mechanism of programmed, locus-specific, somatic genome editing

Authors

Unnati Sonawala, Helen Beasley, Peter Thorpe, ..., John T. Jones, Lida Derevnina, Sebastian Eves-van den Akker

Correspondence

se389@cam.ac.uk

In brief

Sonawala et al. describe how the HYP genes in plant-parasitic nematodes can harbor thousands of alleles. They elucidate the "rules" underlying variation in the hyper-variable domain (HVD) of HYPs and propose that these data are consistent with a mechanism of somatic genome editing, termed HVD editing.

Sonawala et al., 2024, Cell Genomics *4*, 100580
June 12, 2024 © 2024 The Author(s). Published by Elsevier Inc.
https://doi.org/10.1016/j.xgen.2024.100580

CellPress

Cell Genomics

CellPress OPEN ACCESS

Article

A gene with a thousand alleles: The hyper-variable effectors of plant-parasitic nematodes

Unnati Sonawala,[1] Helen Beasley,[1] Peter Thorpe,[2] Kyriakos Varypatakis,[3] Beatrice Senatori,[1] John T. Jones,[3,4] Lida Derevnina,[1] and Sebastian Eves-van den Akker[1,5,*]

[1]The Crop Science Centre, Department of Plant Sciences, University of Cambridge, Cambridge CB2 3EA, UK

[2]The Data Analysis Group, School of Life Sciences, University of Dundee, Dow St., Dundee DD1 5EH, UK

[3]Cell & Molecular Sciences Department, The James Hutton Institute, Invergowrie, Dundee DD2 5DA, UK

[4]School of Biology, University of St Andrews, North Haugh, St Andrews KY16 9ST, UK

[5]Lead contact

*Correspondence: se389@cam.ac.uk

https://doi.org/10.1016/j.xgen.2024.100580

SUMMARY

Pathogens are engaged in a fierce evolutionary arms race with their host. The genes at the forefront of the engagement between kingdoms are often part of diverse and highly mutable gene families. Even in this context, we discovered unprecedented variation in the hyper-variable (HYP) effectors of plant-parasitic nematodes. HYP effectors are single-gene loci that potentially harbor thousands of alleles. Alleles vary in the organization, as well as the number, of motifs within a central hyper-variable domain (HVD). We dramatically expand the HYP repertoire of two plant-parasitic nematodes and define distinct species-specific "rules" underlying the apparently flawless genetic rearrangements. Finally, by analyzing the HYPs in 68 individual nematodes, we unexpectedly found that despite the huge number of alleles, most individuals are germline homozygous. These data support a mechanism of programmed genetic variation, termed HVD editing, where alterations are locus specific, strictly governed by rules, and theoretically produce thousands of variants without errors.

INTRODUCTION

Plant-pathogen interactions result in a co-evolutionary dynamic that drives rapid evolution and diversification of underlying mechanisms. Often, and on both sides, the genes at the forefront of the interaction are part of large, diverse, and highly mutable gene families. Pathogen effectors, and host resistance genes, can be part of some of the most numerous gene families in their respective genomes.[1] Effectors are typically more diverse than other genes in pathogen genomes[2] and, in some cases, are encoded by parts of the genome that mutate rapidly.[3]

Like many pathogen effector genes, HYPs encode nematode proteins that are secreted into the plant, are necessary for infection, have no known homologs, and do not encode any recognized PFAM domains (with the exception of an N-terminal signal peptide for secretion).[4] HYPs, however, are highly unusual, even among effectors. Across three subfamilies, 75 unique genomic sequences (HYP1, $n = 41$; HYP2, $n = 12$; and HYP3, $n = 22$; average length = 888 bp) have been cloned. Irrespective of the subfamily, all cloned HYPs share two continuous strings of coding sequence that are ~95% identical between genes (410 bp at the 5′ and 94 bp at the 3′). Subfamilies are distinguished primarily, although not exclusively, by a subfamily-specific hyper-variable domain (HVD) of unknown function between the conserved regions. Using HYP1 subfamily members as an example, the HVD can encode up to four "motifs," some of which themselves contain variable single- or di-amino acid residues (1.1, 1.2, 1.3, and 1.4). Ignoring the variability within each motif, the 41 unique HYP1 genes contain 17 different organizations of these motifs, with little observable pattern. HYP3s show similar levels of variation (although their HVD contains a different number and organization of an entirely different set of motifs), while HYP2 lacks the HVD altogether.

Regardless of organization, the entire HVD is transcribed as a single exon, in frame with the flanking conserved regions: the variation described is genomic rather than being the result of alternative splicing. Most of the variation within this domain is not just differences in the number of repeats of a particular motif but also differences in the organizations of motifs, which themselves can vary in the sequence that encodes them.

Despite 75 unique HYP genes having been cloned from a population, no individual nematode genome encodes them all. Individuals vary in the types of effector subfamilies that their genomes encode, and individuals vary in their overall number of HYP genes across an order of magnitude. Importantly, this is not several copies of the same gene but several different HYP genes that vary in the organization of their respective HVD.[4] To the best of our knowledge, there is no known mechanism that can account for both the HVD organizations and the apparent gene number variation spanning an order of magnitude between sisters of the same population.

This is an open access article under the CC BY license (http://creativecommons.org/licenses/by/4.0/).

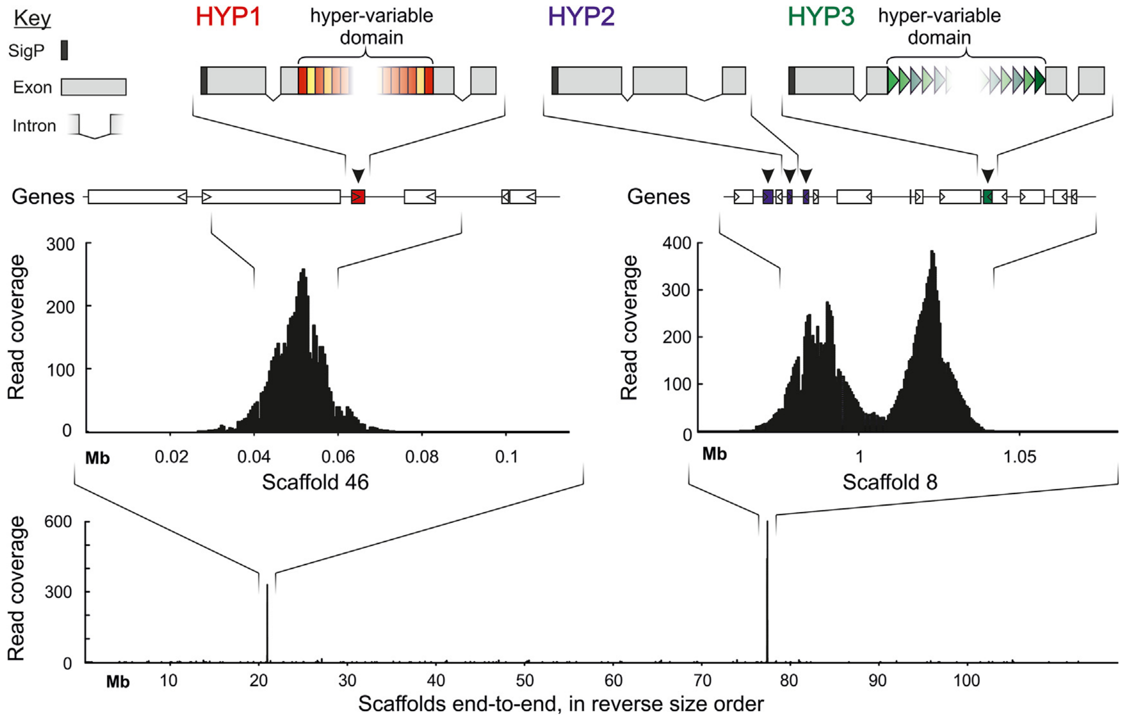

Figure 1. HYP variation is allelic
Schematic of representative HYP1, HYP2, and HYP3 to scale. Hyper-variable domains (HVDs) are indicated by colored blocks (rectangles in shades of reds for HYP1 and triangles in shades of green for HYP3) within the middle of the middle exon (gray blocks). The location of HYPs and adjacent genes is shown on the bottom. Read coverage (black bars) for HYP-containing reads is shown for the HYP1 locus (Scaffold 46, left) and the HYP2- and HYP3-adjacent loci (scaffold 8, right). Substantive coverage is not present on any other scaffolds (bottom).

The genetics underlying HYP effectors has remained elusive because previous genome sequencing attempts pre-dated the discovery of HYPs and used a combination of 76 and 100 bp Illumina reads that are shorter than either HYP conserved region.[5] To understand the genetic basis of variability and stability in the HYP effectors, we sequenced and assembled long-read genomes of two related species. Strikingly, we found single-gene loci for HYP1 and HYP3, indicating that the bewildering diversity of HYPs in fact represents one of the largest allelic series of any organism described: conservatively close to a thousand alleles. We use the expanded HYP repertoire to define the rules underlying HYP "editing" in sufficient detail to simulate HVDs *in silico*. Finally, by analyzing the HYP complement of 68 individual nematodes, we found that despite the huge number of alleles, most individuals are germline homozygous. Hypothetical solutions that explain the juxtaposition of these apparently contradictory phenomena are discussed, perhaps pointing to the underlying cause of the diversity itself.

RESULTS

HYP variation is allelic

To investigate the genetic basis of HYP variation, we needed to first determine the genomic neighborhood of HYP effectors in individual nematodes. Due to the microscopic size of each animal, we employed single-molecule read sequencing (Oxford Nanopore and PacBio) of a population. While we cannot be certain that any two reads came from the same individual (even if they overlap perfectly), we can be absolutely certain that each read came from a single individual. By assigning long HYP-containing single DNA molecules from individuals onto a highly contiguous consensus assembly, based primarily on the non-HYP surrounding sequence, we were able to determine the HYP content of individual nematodes.

Therefore, to provide the necessary high-quality consensus assemblies, the genomes of *Globodera pallida* and *Globodera rostochiensis* were re-sequenced and assembled (see STAR Methods and Table S1). To identify HYP-containing long reads from single *G. pallida* individuals, a hidden Markov model was built for each HYP subfamily. These models were trained on the 75 unique cloned HYP sequences available in NCBI (GenBank: KM206198–KM206272.[4] The parameters were iteratively optimized to accurately identify reads from each subfamily while still maintaining sensitivity for discovering reads with new HYP variants. A total of 251, 334, and 339 unique reads were identified for subfamilies 1, 2, and 3, respectively.

Strikingly, no single read contained more than one HYP, with the exception of HYP2-containing reads. All HYP-containing reads were mapped, in their entirety, to the *de novo* assembly revealing just two, otherwise unremarkable, loci (Figure 1): HYP1-containing reads mapped to *G. pallida* Newton scaffold 46, while HYP2- and HYP3-containing reads mapped to *G. pallida* Newton scaffold 8. Upon closer inspection and in comparison to related isolates and species, a broadly conserved arrangement and the ancestral

state in the *Globodera* were revealed—a single-gene locus for HYP1 and a single-gene locus for HYP3 ~30 kb away from a two- or three-gene locus for HYP2 (Figures 1 and S1).

Taken together, these data show that, strikingly, all of the HYP1 and HYP3 variations observed (which encompass the dominant majority of all HYP variations) are allelic.

The rules of HYP variation

Four degenerate motifs were discovered for HYP1, and two for HYP3, based on our previous Sanger sequencing of cloned HYPs.[4] To determine a fuller extent of HYP variants and the frequency in the population of each, a CRISPR enrichment protocol was developed to selectively enrich for the HYP1 and HYP3 loci, providing thousands of unbiased nanopore reads across each locus (Figure 2). To characterize the variants within a *G. pallida* population, a fraction of the available nanopore reads (the top ~130 highest-quality reads [Phred > 17]) were used in an iterative approach to, as far as possible, classify the HYP motifs within each read (Figures 2A and S2).

In so doing, we were able to re-classify degenerate motifs based on their nucleotide sequence. For example, motif 1.1 ([Y|S][N|E|D]RGGG) can be readily divided into deduced amino acid sequences 1.1.a (YERGGG), 1.1.b (SDRGGG), and 1.1.c (SNRGGG) (Figure S2). The following lists all motifs that can be reliably distinguished in this way in *G. pallida*: 1.1.a (YERGGG), 1.1.b (SDRGGG), 1.1.c (SNRGGG), 1.2.a (RDRGD), 1.2.bc (SDRGD/SDRGE), 1.3 (RDNKRG), and 1.4 (REGGD).

We found that the number and organization of the motifs within the HVD are clearly not random. Most unique HYP1s contain 23 or 24 motifs within their HVD, although this varies greatly (from 1 to 27 motifs). Some motifs occur more frequently than others within unique HYP1s within the entire population (Figure 2B) or within an individual HVD, although this varies greatly: motif 1.1.b is 10 times more common than motif 1.4 overall and, in one case, eight times more common within an HVD.

Interestingly, patterns are clearly evident in the organizations of the motifs. Not all possible adjacent motif pairs were observed, and many of those that were observed were not reciprocal: 1.4 (REGGD) was almost always followed by 1.1.a (YERGGG), but 1.1.a was never followed by 1.4. Exceptions include 1.1.b (SDRGGG) and 1.2.a (RDRGD), which were regularly observed following one another. Strikingly, the most common pair (1.1.a [YERGGG] followed by 1.1.c [SNRGGG]) occurs 180 times more than the least common pair (1.2.bc [SDRGD/SDRGE] followed by 1.1.c [SNRGGG]). Homopolymers were almost never observed: only motifs 1.1.c (SNRGGG) and 1.1.b (SDRGGG) occur in homopolymers, with four 1.1.c being the longest observed. Of all possible organization of motifs in triples (343), we only observed 75, and the distribution is similarly skewed. The top 20 most common triples include all motifs and account for 85% of all observed triples, yet are insufficient to build most HYP1 HVDs with a variable domain >20 (31 out of the 43 HVDs of lengths longer than 20). This suggests that the skewed distribution of observed triples in the population is not due simply to differences between HYPs but rather that most individual HVDs are characterized by common higher-order combinations of motifs interspersed with rare higher-order combinations of motifs.

Zooming out further, patterns coalesce. The HYP1 HVD almost always started (i.e., 54 out of 55 times) and ended (i.e., 47 out of 55 times) with motif 1.1.a (YERGGG). More specifically, HYP1 HVDs often end in the following block of five motifs: 1.4 (REGGD), 1.1.a (YERGGG), 1.1.c (SNRGGG), 1.2.a (RDRGD), and 1.1.a (YERGGG). The probability of each motif in each position (Figure 2C), together with the analysis of adjacent pairs and triples, revealed clear rules to the organization of motifs within the HVD at the scale of the HVD itself. Interestingly, a phylogenetic tree inferred from the non-HVDs of HYP1s (Figure 2D) grouped certain categories of HVDs. This suggests that although the non-HVD is highly conserved, it somehow carries information about the structure of the HVD itself.

Based on just three rules, (1) the possibility of a given motif to follow another one (Figure 2B), (2) the probability of a motif at each position (Figure 2C), and (3) known starts and ends, we can simulate HYP1s with >20 motifs that look "normal." When we simulate a million HYP1 HVDs *in silico*, we re-identify most known organizations and extend the theoretical limit to >73,000 unique variants. This suggests that we have developed a sufficient understanding of the rules of HYP variation to recreate known HYPs *in silico*, and thereby provide a theoretical limit in the tens of thousands, based on extant examples. In contrast, these rules do not apply to the HYP1s of the closely related species *G. rostochiensis* (GrHYP1s). GrHYP1s are shorter (typically 11 or 13 motifs), composed of an almost entirely different set of motifs, and seem to obey a very different set of rules, but they do obey rules nonetheless (Figure S3).

HYP variation is conservatively estimated to exceed a thousand alleles

The theoretical limit of HYP1 variation will massively exceed the true limit because reality is constrained in a multitude of ways that the simulation is not. To estimate the number of alleles in the population, we adopted a population genetics approach. Using the ~130 highest-quality (Phred > q17) nanopore CRISPR-enrichment reads for each species, we identified 55 unique GpHYP1 (*G. pallida* HYP1) variants and 90 unique GrHYP1 variants. Not all variants were equally represented within the populations sequenced. In fact, the distribution was extremely skewed, roughly following Zipf's law: the most common sequence is about twice as common as the next, which itself is about twice as common as the next, etc. The top two most common HYP1s account for 3.6% of unique variants but 50% of all reads, while at the same time, 47 variants each only occur once and account for 85% of unique variants but only 35% of the reads (Figure 3). A similar pattern is observed for GrHYP1s (albeit with an even greater proportion of unique variants—93% of unique variants only occur once (84/90), representing 67% of reads).

We can extrapolate to the total number of alleles in the population by using the frequency distribution of unique sampled alleles in classical species richness calculations. Using the 133 reads alone, the total number of GpHYP1 alleles is estimated to be 235 or 654 using Chao[6] species estimation or the abundance-based coverage estimator (ACE[6,7]), respectively (Figure 3A). The total number of predicted alleles for GrHYP1s is even higher: 1,252 or 1,235 using Chao species estimation or

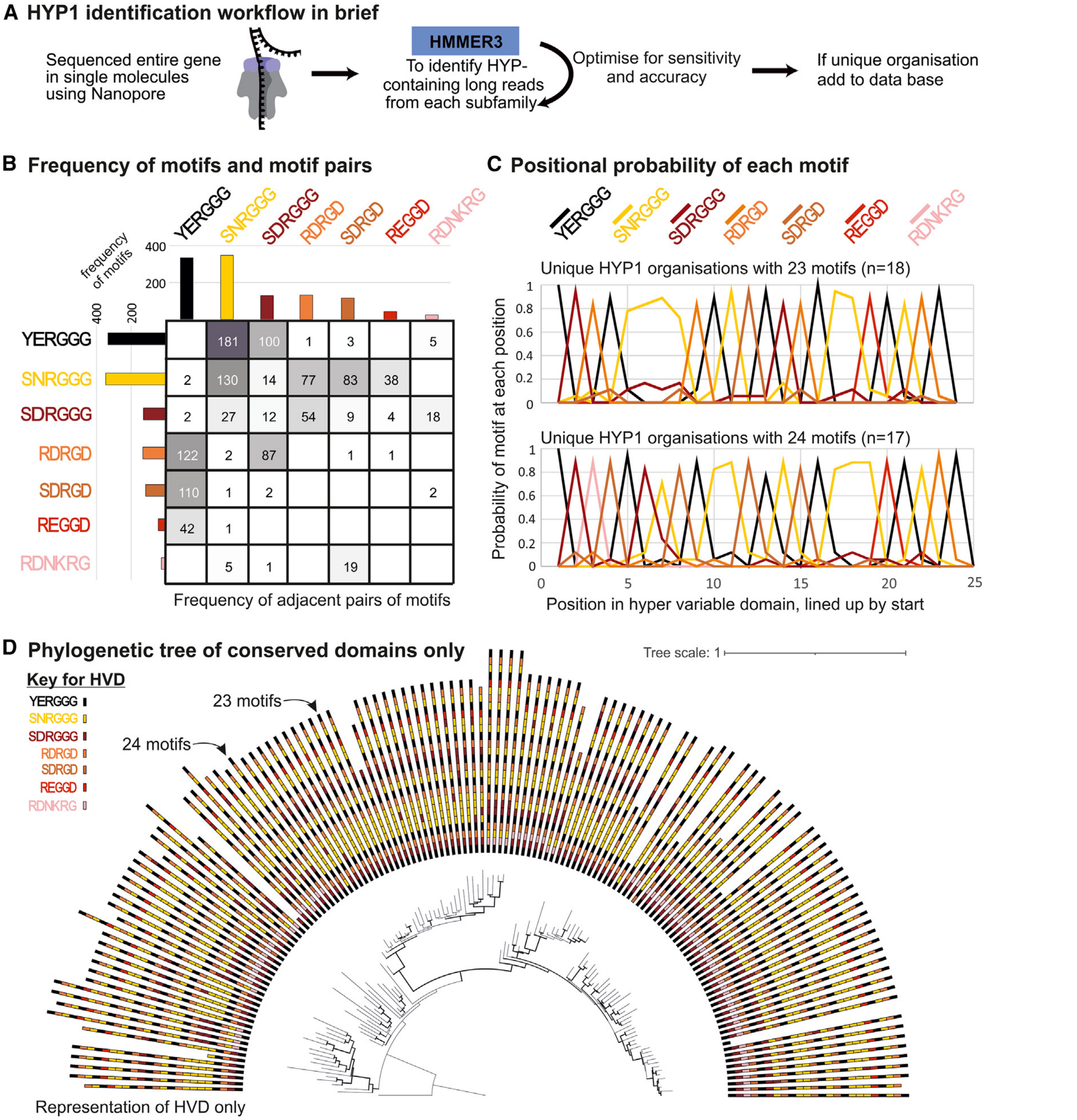

Figure 2. There are rules underlying *G. pallida* HYP1 variation

(A) Brief overview of HYP1 identification workflow (see Figure S2 for full workflow).

(B) Frequency of motif pairs within HYP1 HVDs. Each motif is shown with an amino acid sequence; the y axis of the matrix is position n and the x axis is position n + 1 (YERGGG is never followed by YERGGG, but it is followed by SNRGGG).

(C) The positional probability of each motif at each position for HYP1s with HVDs containing 23 (top) and 24 (bottom) motifs.

(D) A phylogenetic tree inferred from the alignment of the non-HVDs only, displayed with their corresponding HVDs in the outer semi-circle (color coded by motif).

the ACE, respectively (Figure 3B). HYP3 alleles show a similar distribution but are generally less variable than GpHYP1s: 18 were unique out of 130, with estimated population sizes of 31 and 48 for Chao and ACE, respectively (Figure S4). Given that the bulk of HYP variation is HYP1 variation, HYP1s are the focus of this manuscript.

Given that we (1) analyzed a single population of a global pathogen, (2) knowingly ignored all variation outside the HYP domain (Figure 2D), and (3) were conservative in predicting variants in general, these estimates are likely underestimates. Taking these results together, we predict that global HYP1 variation likely exceeds a thousand alleles per locus.

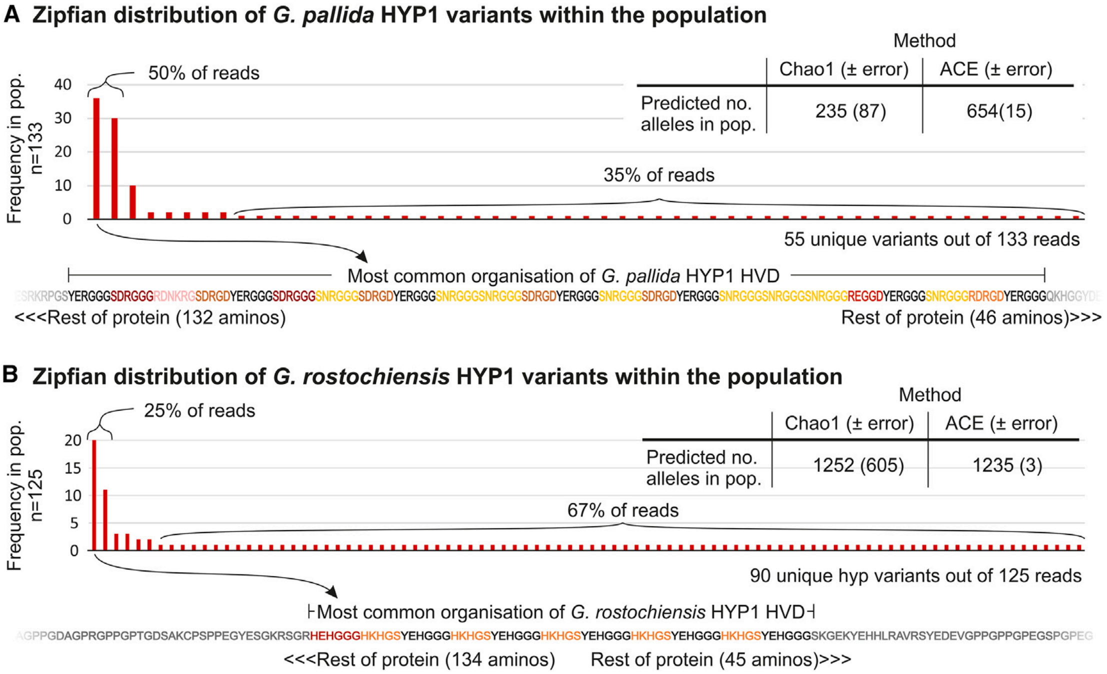

Figure 3. Frequency distribution and population size estimates of HYP1 alleles

(A) *G. pallida* HYP1 and (B) *G. rostochiensis* HYP1 alleles. The graphs show the observed occurrences of each unique HYP1 variant when sampling a population (n = 133 and 125 for *G. pallida* and *G. rostochiensis*, respectively). Inset are two independent species abundance estimates for the total number of alleles in the population based on the sampling. The bottom shows the HVD of the most common variant for each species.

Apparent non-Mendelian inheritance of HYP alleles

To ascertain the contribution of each individual nematode to the overwhelming diversity of HYPs at the population level, we adapted a PCR protocol to reliably function for two amplifications from a second-stage juvenile nematode (J2). From each of 68 individual J2s, the HYP1 and HYP3 loci were amplified (with unique barcode pairs appended to the primers), pooled, and sequenced using PacBio HiFi. In parallel, a series of control amplifications were carried out from known homozygous or heterozygous pools of plasmids containing HYPs.

Consistent with the nanopore data, we found that few HYP variants dominate and that many variants occurred very rarely. As is common for a metagenetic approach in general,[8] within each barcode pair, even homozygous controls, several different HYP alleles could be identified. These could be explained by multiple HYPs in the starting DNA, cross-contamination, mischaracterization of barcodes, and/or sequencing errors (in the gene or the barcode). The controls allowed us to establish a threshold to confidently distinguish between homozygous and heterozygous starting material. As expected, known homozygous controls have most (>90%) of their reads corresponding to a single HYP variant. Heterozygous controls have about half (~40%) of their reads corresponding to a single HYP variant. Analyzing the proportion of reads for each barcode pair contributed by the most common allele allowed us to determine whether the underlying control sample was homozygous or heterozygous without error (Figure 4A). We could not, however, reliably distinguish unequal ratio controls (1:100, 1:500, 1:1,000, 1:2,000, and 1:5,000) from homozygous controls (Figure S5). Therefore, for reads from individual animals of unknown zygosity, we established a threshold of >80% for a likely homozygous sample.

Computing the zygosity of individual J2s revealed a surprising anomaly: most individuals were homozygous (Figure 4A). This result was surprising because the probability of being homozygous decreases as a function of the number of alleles in the population, which we estimated to be over a thousand. Known heterozygous controls do not show homozygosity using these criteria, so we reasoned that this was not an artifact of PCR "selecting" one allele over the other. To calculate the improbability of this result, we made the conservative assumption that the known 55 HYP1 alleles are all that exist and empirically derived the probability of 38/68 individuals being homozygous to be substantially less than one in 10 million: after 10 million simulations of selecting two parental alleles from the population for each of 68 nematodes, the highest number of homozygous individuals was 28/68, which occurred twice (Figure 4B). HYP3 had a similarly large proportion of homozygous individuals (19/45) but seems to be independent from the HYP1 locus (i.e., individuals homozygous for HYP3 are not also preferentially homozygous for HYP1; Figure S6).

Importantly, dominant alleles in those homozygous individuals are not diverse enough to explain the distribution of alleles in the population (Figure 4C). Analyzing the most common allele in each of the 38 HYP1 homozygous individuals revealed just four variants in total, which include the top three most common alleles from the nanopore sequencing (36/133, 30/133, and 10/133) and only one rare allele (1/133). Combining the second

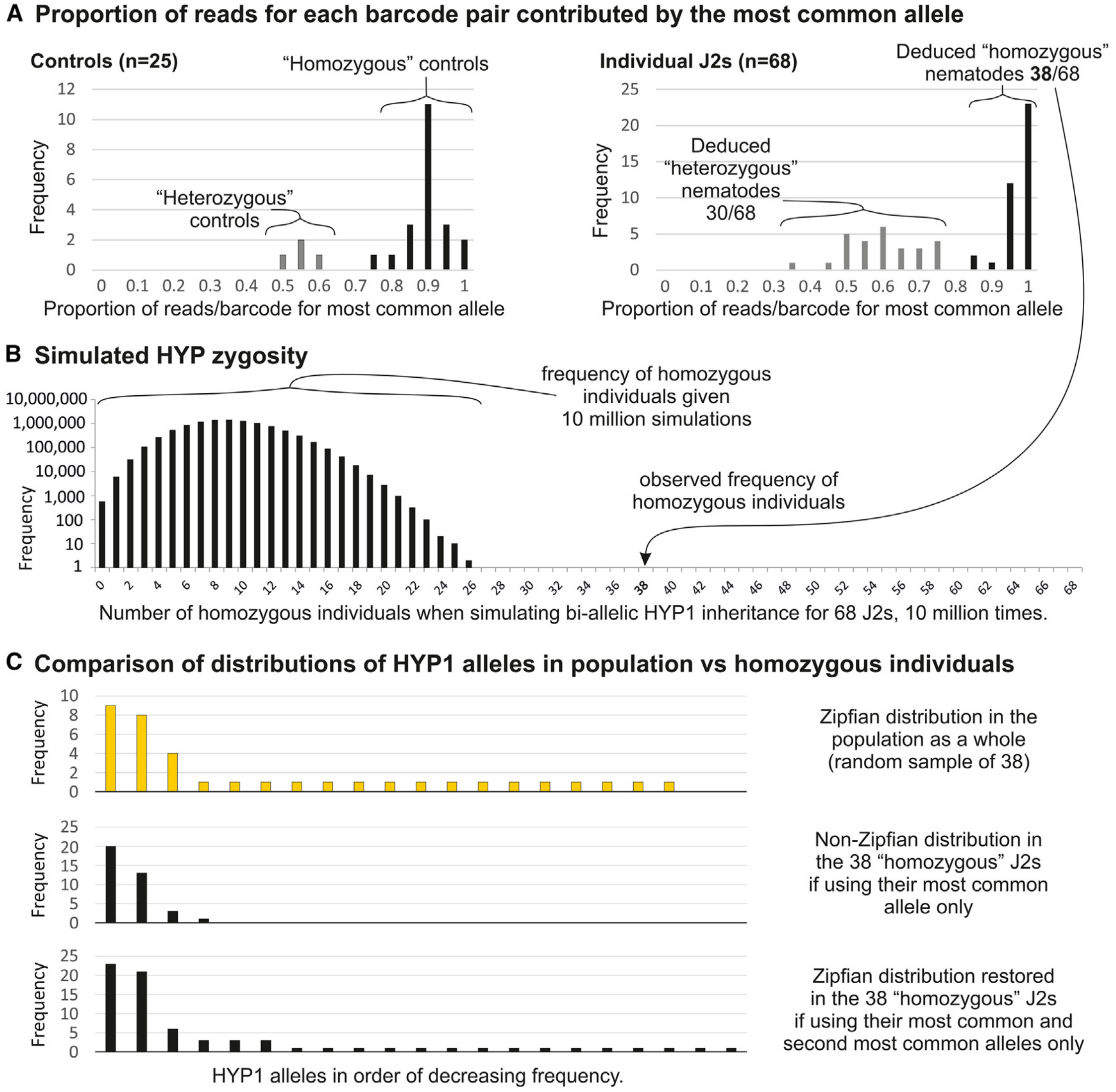

Figure 4. Apparent non-Mendelian inheritance of HYP alleles

(A) Frequency distributions showing the proportion of reads for each barcode pair that is contributed by the most common allele for the 25 control samples (left) and the 68 J2 samples (right). Gray bars indicate heterozygous samples (known for deduced) and black ones indicate homozygous samples (known or deduced).

(B) Frequency of homozygous individuals when simulating bi-allelic HYP1 inheritance for 68 J2s 10 million times.

(C) Frequency distribution of alleles in the whole population (top, yellow), the 38 homozygous J2s if using their most common allele only (middle, black), and the 38 homozygous J2s if using their most common and second most common alleles only (bottom, black).

most common alleles in all 38 homozygous individuals reveals 17 additional variants, 15 of which only occur once in this set. Including the first and second alleles in homozygous individuals is sufficient to recreate the Zipfian distribution observed in the population data.

DISCUSSION

Although we have been extremely conservative at each step when constructing estimates of HYP diversity, an effector with 1,000 alleles is unprecedented in pathology and perhaps in genes in general. HYP allelic variation even exceeds the most widely recognized hyper-variable genes, immunoglobulins. While thousands of immunoglobulin variants have been characterized, and theoretically millions of variants are possible (1×10^7 in humans),[9] V(D)J diversity is achieved by the recombination of segments that exist as multiple copy arrays on the chromosome. By contrast, HYPs are single-copy loci with potentially thousands of alleles.

We can confidently rule out sequencing errors contributing in whole, or even in small part, to both the diversity and nature of the diversity because we have used three sequencing technologies (Nanopore, PacBio, and Sanger), each of which reveals HYP variation, and two species, each of which show considerable but different HYP variation. Importantly, we have found two sets of quite different rules, in that the nature of the variation in

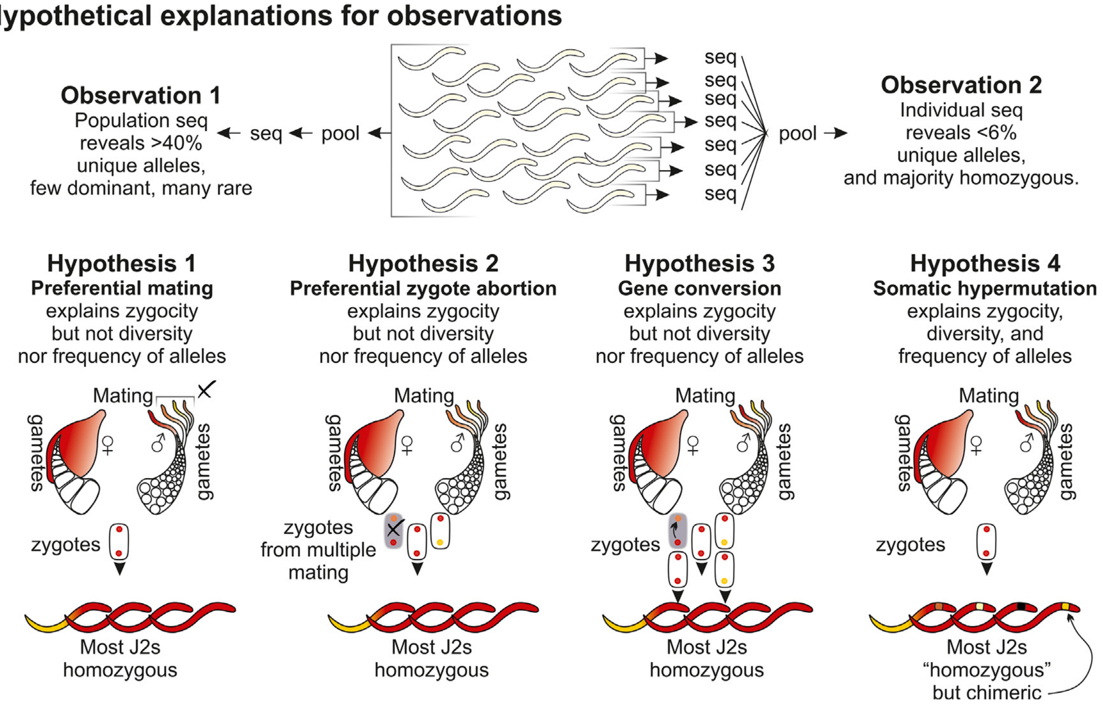

Figure 5. Figure of hypotheses

The primary observations that (1) when sequencing a pool of individuals from a population, we observe many unique alleles (with a long tail of rare alleles) but (2) when we sequence individuals from that same population and pool their dominant allele per individual, we get very few unique alleles (with no long tail of rare alleles). Four potential hypotheses are discussed (preferential mating, preferential zygote abortion, gene conversion, and somatic hypermutation). Hypothesis 4, somatic hypermutation, is the most parsimonious explanation, as it alone explains all phenomena observed.

G. rostochiensis (a pseudo-alternating pattern, Figure S3) is largely different from the nature of the variation in *G. pallida* (shuffling of blocks).

Phylogenetics points to an accumulation of HVD alleles, rather than a contraction of multicopy arrays into a single-copy locus, over evolutionary time. The synteny between *G. pallida* and *G. rostochiensis* suggests that their last common ancestor had all three HYP families. Whether it had the HVDs of today's diversity is unknown, but given that the HVDs of *G. pallida* and *G. rostochiensis* today are markedly different from one another and appear to obey very different sets of rules (Figure S3), we know that the present day complement of HYP alleles in at least one, but perhaps both, of *G. pallida* or *G. rostochiensis* must have arisen since their divergence (i.e., the last 30 million years). Supporting this hypothesis, the close outgroup *Heterodera schachtii* has two HYP-like genes (Hsc_gene_1517 and Hsc_gene_17937) with yet again entirely different HVD structures that resemble neither HYP1s nor HYP3s from either *Globodera* species.[10] Taken together, this suggests that a proto-HYP predated the divergence of *Heterodera* spp. and *Globodera* spp., which is estimated to be some 100 million years ago.

To understand how HYP diversity evolved, i.e., the pathway from one to 1,000 alleles, we need to understand how HYP diversity is created. We intuitively rule out random mutations and subsequent selection because they seem incongruent with the diversity and nature of HYP variants (Figures 2B and 2C). Similarly, we must rule out mechanisms akin, even in small part, to V(D)J recombination because of (1) genetic capital and (2) imprecision. In terms of genetic capital, V(D)J diversity requires a reservoir of multiple copy arrays on the chromosome that HYPs (Figure 1) do not require. Most likely, the genetic capital used to generate new HYP variants comes from within the HVD itself. In terms of imprecision, V(D)J domains are joined by non-homologous end joining, resulting in imprecise joints that contain added nucleotides, contributing to the diversity.[11] The source of HYP diversity is apparently flawless. This is evident from extant HYP genes differing in only the middle of the middle exon without disrupting the open reading frame, despite rearrangements of motifs that differ in length. A mechanism of genome editing that is aware of frame is both unexpected and more akin to guided shuffling/rearranging of the HVD (without inversion) rather than error-prone assembly from a diverse repertoire. We term this phenomenon HVD editing.

Our primary hypothesis on HVD editing comes from the striking observations that (1) sequencing a population reveals many rare alleles and (2) despite potentially thousands of alleles, most individuals appear homozygous for common alleles. We therefore conclude that there must be some mechanism to bias apparent inheritance. Four options are considered: (1) selective mating, (2) abortion of heterozygote zygotes, (3) gene conversion, and (4) post-embryonic hypermutation in the soma (Figure 5). Selective mating and zygote abortion seem highly improbable because the number of alleles could easily be greater than the number of nematodes that may infect a single

plant. Sex resulting in progeny, in either scenario, is deemed to be sufficiently rare to doom the species to extinction.

The gene conversion hypothesis only explains the improbably high proportion of homozygous individuals but fails to account for the lack of diversity between said individuals. Consequently, somatic hypermutation remains the only hypothesis we cannot rule out, as it explains all observed phenomena. Specifically, the Zipfian distribution of allele frequency of the population can only be recapitulated with individual whole-nematode sequencing if we include the second most common allele in homozygous individuals. This may initially seem counterintuitive but is analogous to what would happen if individual whole humans were sequenced and their immunoglobulin diversity analyzed: some rare and re-arranged immunoglobulins, with some dominant alleles that have apparently biased inheritance. This observation would only make sense with the understanding that V(D)J recombination takes place in a small subset of somatic tissues, namely T and B cells. If a similar idea, albeit a different mechanism, gives rise to polyzygotic individuals at the HYP locus from a homozygous progenitor, then it is tempting to think that this would take place in the two cells in which HYPs are expressed, the amphid sheath cells.[4] This would also elegantly explain the observed ratios of HYP variation within the individual and within the population (Figure 5). A programmed difference between the genomes of the germline and the soma, as seen for immunoglobulins, is also important because it means that the mechanism(s) is(are) still happening today—we would be observing an ongoing process, not cataloging what evolution has selected from a greater pool of diversity.

If novel alleles within the individual resemble derivatives of the dominant allele, then this would also favor somatic hypermutation over conversion, i.e., the second most common allele in homozygous individuals would be related to the most common allele in ways that the two most common alleles in heterozygous individuals would not be related to one another. Indeed, the second most common alleles in homozygous individuals are typically shorter than the dominant allele (22/38 J2), whereas for heterozygous individuals, they are not (2/24). Similarly, not all HYPs have all motifs within their HVD, so we would expect second alleles in homozygous individuals, if they were derivatives of the dominant allele, to contain only those motifs present in the dominant allele, but we would not expect the same constraint on the two most common alleles in heterozygous individuals. Indeed, 37/38 second alleles in homozygous individuals contain exclusively motifs present in the dominant allele, whereas the opposite is observed for heterozygous individuals (1/24). Taken together, classical Mendelian inheritance of the locus, coupled with somatic hypermutation in the soma, would explain all observed phenomena.

While determining the teleology of phenomena is often challenging, it is particularly so for HYP variation. Programmed variation is relatively rare but widely distributed across the Tree of Life, including invertebrates and vertebrates.[12] When and why organisms distinguish between germline and somatic genomes vary but can include the "silencing" of germline-active genes in the soma (e.g., programmed DNA elimination[12,13]) or restricting the generation of variation to the soma to balance adaptability in recognizing invading pathogens with genome stability.[11] HYPs are involved in inter-kingdom interactions, expressed during parasitism, and required for full pathogenicity,[4] but in this case, they are deployed by the invading organism. Which aspect of plant biology requires such extreme diversity in the pathogen is not clear, nor whether it is diversity to overcome some aspect of one host or the differences between hosts/host species. The implication would be that the allele you are born with, and presumably the one you pass on to your offspring, is not the one you interact with the plant with. Plant-parasitic nematodes face a very uncertain environment, the host might change every year, and one can imagine strategies evolving that maximize "getting through the year."

Given that pathogens are engaged in a fierce co-evolutionary arms race with their host, it should not be surprising to uncover yet more unusual, but potentially useful, biology (cf. CRISPR,[14] TALENs,[15] and transgenesis[16]). Taking these results together, our working hypothesis is that there is remarkable and potentially useful biology underlying HVD variation, it is active today, it creates variation from genetic capital within the HVD itself, it does so in a subset of the soma (most likely the cells in which they are expressed), it is precisely guided, and it can theoretically produce thousands of such variants without scars. Future work will test these hypotheses.

Limitation of study

In this study, we propose a novel form of locus-specific somatic genome editing, termed HVD editing. While we have an understanding of some rules that apparently underly or constrain the process of generating these edits, the underlying mechanism that gives rise to HYP variation remains unknown. These nematodes are microscopic genetically intractable obligate endoparasites of roots, which makes direct observation of these edits on defined precursor molecules challenging. Much of our analysis is therefore limited by natural variants and allele frequencies in individuals/populations.

STAR★METHODS

Detailed methods are provided in the online version of this paper and include the following:

- KEY RESOURCES TABLE
- RESOURCE AVAILABILITY
  - Lead contact
  - Materials availability
  - Data and code availability
- EXPERIMENTAL MODEL AND STUDY PARTICIPANT DETAILS
- METHOD DETAILS
  - DNA extractions
  - Oxford Nanopore and PacBio sequencing
  - PacBio Hi-Fi sequencing for barcoded PCR from individual nematodes
  - Genome assembly and annotations
  - Finding and filtering HYP containing reads to identify HYP loci
  - CRISPR enrichment of HYP1 and HYP3 loci
  - Motif analysis for HYP1 and HYP3 genes from nanopore and PacBio HiFi sequencing
  - Simulating HYP HVDs *in silico*
- QUANTIFICATION AND STATISTICAL ANALYSIS
  - Zygosity
- ADDITIONAL RESOURCES

SUPPLEMENTAL INFORMATION

Supplemental information can be found online at https://doi.org/10.1016/j.xgen.2024.100580.

ACKNOWLEDGMENTS

Work on plant-parasitic nematodes at the University of Cambridge is supported by DEFRA license 125034/359149/3 and funded by BBSRC grants BB/R011311/1, BB/S006397/1, and BB/X006352/1; a Leverhulme grant RPG-2023-001; and a UKRI Frontier Research Grant EP/X024008/1. K.V. was funded through an AHDB PhD award. J.T.J. receives funding from the Scottish Government Rural and Environmental Services Division.

AUTHOR CONTRIBUTIONS

U.S. performed the experiments and data analysis; H.B. and B.S. prepared the biological material; and P.T., K.V., and J.T.J. were responsible for the sequencing and assembly of the genome of *G. pallida* Newton. S.E.-v.d.A. and U.S. drafted the manuscript. All authors read and approved the final manuscript.

DECLARATION OF INTERESTS

The authors declare no competing interests.

Received: November 14, 2023
Revised: March 5, 2024
Accepted: May 6, 2024
Published: May 29, 2024

REFERENCES

1. Tang, D., Jia, Y., Zhang, J., Li, H., Cheng, L., Wang, P., Bao, Z., Liu, Z., Feng, S., Zhu, X., et al. (2022). Genome evolution and diversity of wild and cultivated potatoes. Nature *606*, 535–541.
2. Eves-van den Akker, S., Laetsch, D.R., Thorpe, P., Lilley, C.J., Danchin, E.G.J., Da Rocha, M., Rancurel, C., Holroyd, N.E., Cotton, J.A., Szitenberg, A., et al. (2016). The genome of the yellow potato cyst nematode, Globodera rostochiensis, reveals insights into the basis of parasitism and virulence. Genome Biol. *17*, 124.
3. Rouxel, T., Grandaubert, J., Hane, J.K., Hoede, C., van de Wouw, A.P., Couloux, A., Dominguez, V., Anthouard, V., Bally, P., Bourras, S., et al. (2011). Effector diversification within compartments of the Leptosphaeria maculans genome affected by Repeat-Induced Point mutations. Nat. Commun. *2*, 202.
4. Eves-van den Akker, S., Lilley, C.J., Jones, J.T., and Urwin, P.E. (2014). Identification and characterisation of a hyper-variable apoplastic effector gene family of the potato cyst nematodes. PLoS Pathog. *10*, e1004391.
5. Cotton, J.A., Lilley, C.J., Jones, L.M., Kikuchi, T., Reid, A.J., Thorpe, P., Tsai, I.J., Beasley, H., Blok, V., Cock, P.J.A., et al. (2014). The genome and life-stage specific transcriptomes of Globodera pallidaelucidate key aspects of plant parasitism by a cyst nematode. Genome Biol. *15*, R43.
6. O'hara, R.B. (2005). Species richness estimators: how many species can dance on the head of a pin? J. Anim. Ecol. *74*, 375–386.
7. Chiu, C.-H., Wang, Y.-T., Walther, B.A., and Chao, A. (2014). An improved nonparametric lower bound of species richness via a modified good–turing frequency formula. Biometrics *70*, 671–682.
8. Eves-van den Akker, S., Lilley, C.J., Reid, A., Pickup, J., Anderson, E., Cock, P.J.A., Blaxter, M., Urwin, P.E., Jones, J.T., and Blok, V.C. (2015). A metagenetic approach to determine the diversity and distribution of cyst nematodes at the level of the country, the field and the individual. Mol. Ecol. *24*, 5842–5851.
9. Schroeder, H.W., Jr. (2006). Similarity and divergence in the development and expression of the mouse and human antibody repertoires. Dev. Comp. Immunol. *30*, 119–135.
10. Siddique, S., Radakovic, Z.S., Hiltl, C., Pellegrin, C., Baum, T.J., Beasley, H., Bent, A.F., Chitambo, O., Chopra, D., Danchin, E.G.J., et al. (2022). The genome and lifestage-specific transcriptomes of a plant-parasitic nematode and its host reveal susceptibility genes involved in trans-kingdom synthesis of vitamin B5. Nat. Commun. *13*, 6190.
11. Chi, X., Li, Y., and Qiu, X. (2020). V(D)J recombination, somatic hypermutation and class switch recombination of immunoglobulins: mechanism and regulation. Immunology *160*, 233–247.
12. Wang, J., Gao, S., Mostovoy, Y., Kang, Y., Zagoskin, M., Sun, Y., Zhang, B., White, L.K., Easton, A., Nutman, T.B., et al. (2017). Comparative genome analysis of programmed DNA elimination in nematodes. Genome Res. *27*, 2001–2014.
13. Gonzalez de la Rosa, P.M., Thomson, M., Trivedi, U., Tracey, A., Tandonnet, S., and Blaxter, M. (2021). A telomere-to-telomere assembly of Oscheius tipulae and the evolution of rhabditid nematode chromosomes. G3 (Bethesda) *11*, jkaa020. https://doi.org/10.1093/g3journal/jkaa020.
14. Jinek, M., Chylinski, K., Fonfara, I., Hauer, M., Doudna, J.A., and Charpentier, E. (2012). A programmable dual-RNA-guided DNA endonuclease in adaptive bacterial immunity. Science *337*, 816–821.
15. Boch, J., Scholze, H., Schornack, S., Landgraf, A., Hahn, S., Kay, S., Lahaye, T., Nickstadt, A., and Bonas, U. (2009). Breaking the code of DNA binding specificity of TAL-type III effectors. Science *326*, 1509–1512.
16. Joos, H., Timmerman, B., Montagu, M.V., and Schell, J. (1983). Genetic analysis of transfer and stabilization of Agrobacterium DNA in plant cells. EMBO J. *2*, 2151–2160.
17. Ruan, J., and Li, H. (2020). Fast and accurate long-read assembly with wtdbg2. Nat. Methods *17*, 155–158.
18. Koren, S., Walenz, B.P., Berlin, K., Miller, J.R., Bergman, N.H., and Phillippy, A.M. (2017). Canu: scalable and accurate long-read assembly via adaptive k-mer weighting and repeat separation. Genome Res. *27*, 722–736.
19. Roach, M.J., Schmidt, S.A., and Borneman, A.R. (2018). Purge Haplotigs: allelic contig reassignment for third-gen diploid genome assemblies. BMC Bioinf. *19*, 460.
20. Laetsch, D.R., and Blaxter, M.L. (2017). BlobTools: Interrogation of genome assemblies. F1000Res. *6*, 1287.
21. Lam, K.-K., LaButti, K., Khalak, A., and Tse, D. (2015). FinisherSC: a repeat-aware tool for upgrading de novo assembly using long reads. Bioinformatics *31*, 3207–3209.
22. Boetzer, M., and Pirovano, W. (2014). SSPACE-LongRead: scaffolding bacterial draft genomes using long read sequence information. BMC Bioinf. *15*, 211.
23. Kammonen, J.I., Smolander, O.-P., Paulin, L., Pereira, P.A.B., Laine, P., Koskinen, P., Jernvall, J., and Auvinen, P. (2019). gapFinisher: A reliable gap filling pipeline for SSPACE-LongRead scaffolder output. PLoS One *14*, e0216885.
24. Walker, B.J., Abeel, T., Shea, T., Priest, M., Abouelliel, A., Sakthikumar, S., Cuomo, C.A., Zeng, Q., Wortman, J., Young, S.K., and Earl, A.M. (2014). Pilon: an integrated tool for comprehensive microbial variant detection and genome assembly improvement. PLoS One *9*, e112963.
25. Li, H. (2018). Minimap2: pairwise alignment for nucleotide sequences. Bioinformatics *34*, 3094–3100.
26. Li, H., and Durbin, R. (2009). Fast and accurate short read alignment with Burrows-Wheeler transform. Bioinformatics *25*, 1754–1760.
27. Thorpe, P., Escudero-Martinez, C.M., Cock, P.J.A., Eves-van den Akker, S., and Bos, J.I.B. (2018). Shared transcriptional control and disparate gain and loss of aphid parasitism genes. Genome Biol. Evol. *10*, 2716–2733.
28. Haas, B. (2007). TransposonPSI: An Application of PSI-Blast to Mine (Retro-) Transposon ORF Homologies (Broad Institute).

29. Ellinghaus, D., Kurtz, S., and Willhoeft, U. (2008). LTRharvest, an efficient and flexible software for de novo detection of LTR retrotransposons. BMC Bioinf. *9*, 18.

30. Gremme, G., Steinbiss, S., and Kurtz, S. (2013). GenomeTools: a comprehensive software library for efficient processing of structured genome annotations. IEEE/ACM Trans. Comput. Biol. Bioinform. *10*, 645–656.

31. Quinlan, A.R. (2014). BEDTools: The Swiss-army tool for genome feature analysis. Curr. Protoc. Bioinformatics *47*, 11.12.1–11.12.34.

32. Dobin, A., Davis, C.A., Schlesinger, F., Drenkow, J., Zaleski, C., Jha, S., Batut, P., Chaisson, M., and Gingeras, T.R. (2013). STAR: ultrafast universal RNA-seq aligner. Bioinformatics *29*, 15–21.

33. Danecek, P., Bonfield, J.K., Liddle, J., Marshall, J., Ohan, V., Pollard, M.O., Whitwham, A., Keane, T., McCarthy, S.A., Davies, R.M., and Li, H. (2021). Twelve years of SAMtools and BCFtools. GigaScience *10*, giab008. https://doi.org/10.1093/gigascience/giab008.

34. Haas, B.J., Papanicolaou, A., Yassour, M., Grabherr, M., Blood, P.D., Bowden, J., Couger, M.B., Eccles, D., Li, B., Lieber, M., et al. (2013). De novo transcript sequence reconstruction from RNA-seq using the Trinity platform for reference generation and analysis. Nat. Protoc. *8*, 1494–1512.

35. Hoff, K.J., Lange, S., Lomsadze, A., Borodovsky, M., and Stanke, M. (2016). BRAKER1: Unsupervised RNA-Seq-Based Genome Annotation with GeneMark-ET and AUGUSTUS. Bioinformatics *32*, 767–769.

36. Lukashin, A.V., and Borodovsky, M. (1998). GeneMark.hmm: new solutions for gene finding. Nucleic Acids Res. *26*, 1107–1115.

37. Pertea, M., Pertea, G.M., Antonescu, C.M., Chang, T.-C., Mendell, J.T., and Salzberg, S.L. (2015). StringTie enables improved reconstruction of a transcriptome from RNA-seq reads. Nat. Biotechnol. *33*, 290–295.

38. Phanstiel, D.H., Boyle, A.P., Araya, C.L., and Snyder, M.P. (2014). Sushi.R: flexible, quantitative and integrative genomic visualizations for publication-quality multi-panel figures. Bioinformatics *30*, 2808–2810.

39. Wickham, H. (2016). ggplot2: Elegant Graphics for Data Analysis (Springer).

40. McKenna, A., and Shendure, J. (2018). FlashFry: a fast and flexible tool for large-scale CRISPR target design. BMC Biol. *16*, 74.

41. Gilpatrick, T., Lee, I., Graham, J.E., Raimondeau, E., Bowen, R., Heron, A., Downs, B., Sukumar, S., Sedlazeck, F.J., and Timp, W. (2020). Targeted nanopore sequencing with Cas9-guided adapter ligation. Nat. Biotechnol. *38*, 433–438.

42. De Coster, W., D'Hert, S., Schultz, D.T., Cruts, M., and Van Broeckhoven, C. (2018). NanoPack: visualizing and processing long-read sequencing data. Bioinformatics *34*, 2666–2669.

43. Dixon, P. (2003). VEGAN, a package of R functions for community ecology. J. Veg. Sci. *14*, 927–930.

STAR★METHODS

KEY RESOURCES TABLE

REAGENT or RESOURCE	SOURCE	IDENTIFIER
Chemicals, peptides, and recombinant proteins		
CRISPR-Cas9 tracrRNA	IDT	Cat #1072532
AMPure XP beads	Beckman Coulter	Cat #A63882
Shrimp Alkaline Phosphatase (rSAP)	NEB	Cat #M0371S
Critical commercial assays		
Ligation Sequencing Kit	Oxford Nanopore	SQK-LSK109, SQK-LSK112
Flow Cell Wash Kit	Oxford Nanopore	WSH003/WSH004
BluePippin High Pass size-selection kit	Sage Science	PAC20KB (with S1 and U1 markers)
KOD Xtreme Hot Start DNA Polymerase	Sigma Aldrich	Cat #71975-3
Deposited data		
Whole genome nanopore sequencing of *G. pallida* 'Lindley' and *G. rostochiensis* '(raw reads and genome)	This paper	GenBank: PRJNA1078841
Targeted nanopore sequencing of HYP loci from *G. pallida* and *G. rostochiensis*	This paper	GenBank: PRJNA1078841
PacBio amplicon sequencing of HYP amplicons from single nematodes	This paper	GenBank: PRJNA1078841
Whole genome sequencing of *G. pallida* 'Newton'	This paper	GenBank: PRJNA702104
Scripts used for motif analysis of HYPs	This paper	Zenodo: https://doi.org/10.5281/zenodo.11108598
Scripts for simulation of HVD in silico and zygosity calculations	This paper	Zenodo: https://doi.org/10.5281/zenodo.11235347
Scripts for testing gene calls	This paper	Zenodo: https://doi.org/10.5281/zenodo.11109054
Experimental models: Organisms/strains		
Globodera pallida	James Hutton Institute, UK	Taxon ID: 36090
Globodera rostochiensis 'Ro1'	James Hutton Institute, UK	Taxon ID: 31243
Oligonucleotides		
Barcoded oligonucleotides for HYP1 and HYP3 loci, *G. pallida*	This paper	Table S3
crRNA for targeted HYP1 and HYP3 Nanopore sequencing	This paper	Table S3
Software and algorithms		
wtdbg2	Ruan and Li[16]	https://github.com/ruanjue/wtdbg2
Canu	Koren et al.[17]	https://github.com/marbl/canu
Purge haplotigs	Roach et al.[18]	https://bitbucket.org/mroachawri/purge_haplotigs
BlobTools	Laetsch and Blaxter[19]	https://github.com/DRL/blobtools
minialign		https://github.com/ocxtal/minialign
FinisherSC	Lam et al.[20]	https://github.com/kakitone/finishingTool
SSPACE-LongRead	Boetzer and Pirovano[21]	https://github.com/Runsheng/sspace_longread
gapFinisher	Kammonen et al.[22]	https://github.com/kammoji/gapFinisher
Pilon	Walker et al.[23]	https://github.com/broadinstitute/pilon
minimap2	Li H.[24]	https://github.com/lh3/minimap2
BWA-MEM	Li and Durbin[25]	https://github.com/lh3/bwa

(Continued on next page)

Continued

REAGENT or RESOURCE	SOURCE	IDENTIFIER
ggplot2	Wickham H.[26]	https://cran.r-project.org/web/packages/ggplot2/index.html
vegan (R package)	Dixon[27]	https://cran.r-project.org/web/packages/vegan/index.html
TrasnposonPSI	Haas B.[28]	https://transposonpsi.sourceforge.net/
LTRHarvest	Ellinghaus et al.[29]	https://github.com/genometools/genometools
Genometools	Gremme et al.[30]	https://github.com/genometools/genometools
bedtools	Quinlan[31]	https://bedtools.readthedocs.io/
Guppy	Oxford Nanopore	https://community.nanoporetech.com/downloads
STAR	Dobin[5]	https://github.com/alexdobin/STAR
Trinity	Haas et al.[32]	N/A
BRAKER	Hoff et al.[33]	N/A
GeneMark-ET	Lukashin and Borodovsky[34]	N/A
Funannotate		https://zenodo.org/records/2604804
Stringtie	Pertea et al.[35]	https://ccb.jhu.edu/software/stringtie
SAMtools	Danecek et al.[36]	https://github.com/samtools/samtools
Scripts used for motif analysis of HYPs	This paper	https://doi.org/10.5281/zenodo.11108598
Scripts for simulation of HVD in silico and zygosity calculations	This paper	https://doi.org/10.5281/zenodo.11235347
Scripts for testing gene calls	This paper	https://doi.org/10.5281/zenodo.11109054

RESOURCE AVAILABILITY

Lead contact

Further information and requests for resources and reagents should be directed to and will be fulfilled by the lead contact, Sebastian Eves-van den Akker (se389@cam.ac.uk).

Materials availability

This study did not generate new unique reagents.

Data and code availability

WGS raw reads and genomes of *G. rostochiensis* 'Ro1' and *G.pallida* 'Lindley', raw reads for targeted sequencing of HYPs, and raw reads from amplicon sequencing of HYPs from individual nematodes are available under GenBank: PRJNA1078841.

Data and assembly for *G. pallida* 'Newton' are available under BioProject number PRJNA702104.

Scripts used for motif analysis of HYPs are available from https://github.com/unnatisonawala/HYPervariable_HYPs – all of which are additionally archived under a stable Zenodo DOI: https://doi.org/10.5281/zenodo.11108598.

Scripts for simulation of HVD *in silico* are available from https://github.com/sebastianevda/HYP/tree/main/Simulate_HVDs along with scripts for zygosity calculations https://github.com/sebastianevda/HYP/tree/main/Zygosity – all of which are additionally archived under a stable Zenodo DOI: https://doi.org/10.5281/zenodo.11235347.

Scripts for testing gene calls are available from https://github.com/peterthorpe5/public_scripts/tree/master/gene_model_testing – all of which are additionally archived under a stable Zenodo DOI: https://doi.org/10.5281/zenodo.11109054.

EXPERIMENTAL MODEL AND STUDY PARTICIPANT DETAILS

The experiments described herein used the potato cyst nematodes *Globodera pallida* populations "Lindley" and "Newton", and *G. rostochiensis* population "Ro1" – all obtained from the James Hutton Institute. All experiments were performed on the second stage Juveniles (J2) with the exception of the amplicon sequencing which was additionally performed on males, females, and cysts.

METHOD DETAILS

DNA extractions

A pellet of freshly hatched J2s was flash-frozen in liquid nitrogen. Between 30,000 and 50,000 J2s were used for high-molecular weight (HMW) DNA extraction, resulting in ~3.5-5μg of DNA. Briefly, a pellet of frozen J2s was homogenised in 20 μL of lysis buffer (0.1M Tris at pH 8.0, 0.5M NaCl, 50mM EDTA and 1%SDS) using a micropestle. Additional 140 μL of lysis buffer and 40 μL of proteinase K (20 mg/ml, Promega, Cat. No. MC5005) were added, and incubated at 55°C for 18 h. 10μL of RNAseA (10 mg/ml, Thermo Scientific, Cat. No. EN0531) was added, mixed gently and incubated for 5 min at room temperature. Equal volume of phenol/chloroform/isoamyl alcohol was added to the lysed J2s and mixed by rotating on a hula mixer for 15 min. Post centrifugation, the aqueous solution was collected in a fresh tube and the above step was repeated by adding a 10mM Tris-HCl (pH 8.5) buffer to the organic phase. The DNA was further purified by using an equal volume of chloroform/isoamyl alcohol (24:1) for one round of back extraction. DNA was precipitated by adding NH_4OAc (0.75M), glycogen (20 μg) and 2.5 volume of 100% ethanol. The DNA was centrifuged at 4°C for 20 min. The resulting pellet was washed two times with 80% ethanol, air dried, and resuspended in 10 mM Tris-HC (pH 8.5). DNA in aqueous solution was handled using wide-bore tips and extraction was performed using low-retention microcentrifuge tubes. DNA amount was measured using Qubit fluorometer (Invitrogen) and its purity was assessed by measuring the absorbance ratios using NanoDrop 1000 spectrophotometer (ThermoFisher Scientific).

For DNA from single nematodes, extractions were performed in worm lysis buffer (WLB) (50mM KCl, 10mM Tris (pH 8.3), 2.5mM $MgCl_2$, 0.45% NP-40 (IGEPAL), and 0.45% Tween 20) with Proteinase K (20 mg/ml, Promega, Cat. No. MC5005) added just before use. Single nematodes were placed in PCR strip tubes containing 5μL of WLB. They were subjected to three rounds of freeze-thaw using flash-freezing in liquid nitrogen. An additional 5μL of WLB containing Proteinase K (12μL of Proteinase K in 88μL of WLB) was added to each tube. The nematodes were digested at 65°C for 90 min followed by inactivation of Proteinase K at 95°C for 15 min.

Oxford Nanopore and PacBio sequencing

Nanopore sequencing libraries were prepared using the Ligation Sequencing kit (SQK-LSK109, Oxford Nanopore) following the manufacturer's protocol (version: GDE_9063_v109_revQ_14Aug2019) with the following modifications: Incubation with and elution from AMPure XP beads (Beckman Coulter, Cat. No. A63882) was extended to 30 min each. Incubation for ligating adapters was extended to 2 h at room temperature. Potato cyst nematode DNA appears to block the pores in the flowcells within 12 h of running on a MinIon, resulting in fewer reads. Hence, when possible, two libraries were prepared and Flow Cell Wash Kit (WSH003/WSH004, Oxford Nanopore) was used to regenerate the pores after the first run before loading the second library. The second library was stored at 4°C until loading. One flowcell run was used to generate sequencing data for *G. pallida* 'Lindley' and three flowcell runs were used for *G. rostochiensis* 'Ro1'; two of these runs were from DNA size-selected using 15 kbp and 30 kbp cutoff points, respectively. Size selection was performed using BluePippin and the associated PAC20KB kit (Sage Science) according to the manufacturer's protocol. Sequencing reads were basecalled using the latest version of Guppy at the time of sequencing (versions: 3.3.0+ef22818 for run 1, 3.4.4 + a296acb for run 2 and 4.0.11 + f1071ce for run 3 of *G.rostochiensis* libraries, and version 3.4.4 + a296acb for *G.pallida* library). Guppy basecaller was used with the following config file for high accuracy basecalling: dna_r9.4.1_450bps_hac.cfg. For PacBio sequencing of *G. pallida* 'Newton', 20Kb shear was performed and size selection was performed using a Blue Pippin (Sage Science) with an 8Kb cut-off point. The library was sequenced on Pacific Biosciences Sequel instrument.

PacBio Hi-Fi sequencing for barcoded PCR from individual nematodes

Randomised barcodes of 9-10bp, with a minimum Hamming distance of five, were generated using https://github.com/audy/barcode-generator. PCR using barcoded primers (Table S3) was performed using KOD Xtreme Hot Start DNA Polymerase (Cat. No. 71975-3) using the following reaction mixture: 25μL 2X Xtreme buffer, 10μL dNTPs (2mM), 1.5μL each of forward and reverse primers at 10μM, 6μL of nuclease-free water, and 5μL of single nematode DNA (extracted as described earlier). The PCR was performed using the following conditions: initial denaturation at 94°C for 2 min; 40 cycles of 98°C for 10 s, 55°C for 30 s and 68°C for 1 min 30 s for HYP1 gene (and 2 min for HYP3 gene); final extension at 68°C for 7 min. The PCR products were cleaned using Monarch PCR & DNA cleanup kit (Cat. No. T1030L), and pooled roughly by normalising for amounts based on the intensity of their band visualised during gel electrophoresis. ~4μg of the pooled amplicons were used for PacBio HiFi sequencing on a Sequel II instrument.

Genome assembly and annotations

Genomes from Nanopore reads (*G. rostochiensis* 'Ro1' and G.pallida 'Lindley') were assembled using wtdbg2.[17] In addition to the preset -x ont, the following additional parameters were used for *G. pallida* 'Lindley': -S 1 and -A and the following for *G. rostochiensis* 'Ro1': -S 1 -A -L 10000. PacBio subreads generated from four flow cells for *G. pallida* 'Newton' were concatenated and error-corrected using Canu (v1.7[18]) with the following additional parameters: correctedErrorRate = 0.15 corOutCoverage = 300. Canu error-corrected reads were used to generate the final assembly using wtdbg2 with the following parameters: -L 4000 and -p 19.

For all genome assemblies, purge haplotigs pipeline was used to remove duplicated contigs from the primary assembly.[19] The primary contig assemblies were subsequently assessed for contamination using BlobTools version 1.0.[20] Briefly, reads were mapped to the assembly using minialign version 0.5.2 (https://github.com/ocxtal/minialign) to determine the coverage of the assembled

contigs. The contigs were BLASTn searched against GenBank nt database with taxonomic information in the tabular output. Contigs were then taxonomically classified based on the weight of the BLAST hits. Identified contaminant contigs were removed thus, yielding a contamination free final unpolished contig level assembly. The assembly was further improved using FinisherSC.[21] SSPACE-LongRead[21,22] was used to scaffold the contigs using the following parameters: -k 1 -o 1000 -L 30 for Nanopore assemblies and -g 500 -L 10 -o 100 -k 1 for the PacBio assembly. Gaps in the assembly were filled using gapFinisher.[23] The scaffolds were further polished and corrected using five rounds of Pilon[24] using both raw reads from Nanopore/PacBio sequencing and short paired-end reads from Illumina HiSeq 2000. Following short reads accessions were downloaded from ENA and used for assembly correction (https://www.ebi.ac.uk/): ERR114517 and ERR114518 for *G. pallida* 'Lindley' and ERR114519, ERR123958, and ERR114520 for *G. rostochiensis* 'Ro1'. minimap2[25] was used to map nanopore reads and BWA-MEM[26] was used to map the short reads to the genome assembly.

Transposon prediction, and hard and soft repetitive genome masking, was performed as described in.[27] Briefly, Repeatmodeler (version DEV) was used to identify repetitive regions. The resulting repetitive elements were masked using RepeatMasker along with RepBaseRepeatMaskerEdition-20170127 models. To additionally identify transposons, TransposonPSI (version 08222010)[28] and LTRharvest version 1.5.9[29] from Genometools[30] was used. Finally, bedtools[31] version 2.27.1 was used to softmask the genome for gene prediction. RNAseq data from the relevant species[2,5] was quality (Q30) trimmed using trimmomatic, allowing a minimum read length of 67, and mapped to the final genomes using STAR (version 020201)[32] with the following parameters –outFilterMismatchNmax 7 –outFilterMultimapNmax 5. The resulting bam files were merged, sorted and indexed using SAMtools.[33] The sorted bam was used to perform a *de novo* genome-guided RNAseq assembly using Trinity version 2.8.4[34] with the additional parameters (–genome_guided_max_intron 15000 –genome_guided_min_coverage 5). The softmasked genome along with the RNAseq mapped.bam file was subjected to unsupervised gene prediction using BRAKER version 2[35] with the additional Augustus parameters –protein = on –start = on –stop = on –cds = on –introns = on –noInFrameStop = true –genemodel = complete and the additional BRAKER parameters –filterOutShort –UTR = on. The resulting BRAKER predicted GFF file and the GeneMark-ET[36] GFF files were passed to Funannotate (DOI: https://doi.org/10.5281/zenodo.2604804) with the weighting of 5 and 1 respectively (out of 10). DIAMOND_BLASTP search against Swiss-prot was also given as evidence during the prediction phase. The genome-guided assembly was given to Funannotate which runs PASA, the resulting PASA models were given a score of 6 (out of 10) in the Evidence modeller stage. The update stage of Funannotate refines the introns, exon, start, stops using a combination of PASA and Stringtie.[37] Gene calls were tested with https://github.com/peterthorpe5/public_scripts/tree/master/gene_model_testing - https://doi.org/10.5281/zenodo.11109054.

Finding and filtering HYP containing reads to identify HYP loci

Previously cloned HYP genes were used to build an HMM model for HYP1 (Data S1) HYP2 (Data S2), and HYP3 (Data S3). HYP containing long pacbio raw reads were identified by using HMMR3. Multiple evalues were tested to ensure that the reads did not overlap between subfamilies. Evalue of 1e−100 was found to be optimal. These reads were then mapped back to the genome using minimap2 (minimap2 -ax map-pb –secondary = no). The aligned reads were converted to a bedgraph using bamCoverage function from the deepTools package (using -bs 10000). The plots were plotted using the plotBedgraph() function from the Sushi package in R.[38] Schematic gene maps were drawn using the ggplot2[39] extension gggenes.

CRISPR enrichment of HYP1 and HYP3 loci

gRNA design and duplex assembly

crRNAs (IDT) were used with tracrRNA (IDT) to form a functional gRNA duplex. FlashFry[40] was used to identify and design crRNAs. Guides were designed to flank HYP1 and HYP3 genes for *G. pallida* and *G. rostochiensis* using the PacBio *G.pallida* (Newton) assembly and Nanopore - corrected *G. rostochiensis* (Ro1) assembly. Candidate guides were filtered using these following parameters: dangerous_in_genome = "IN_GENOME = 1", dangerious_GC and dangerous_polyT = NONE and baseDiffToClosestHit ≥ 3. Candidate guides were then validated using *in vitro* Cas9 cleavage assay, and guides with the highest cleavage efficiency were used for enrichment (Table S2).

SQK-LSK112 ligation sequencing kit was used on MinIon flow cells (version R10.4.1). Libraries were prepared by adapting the Cas9-targeted sequencing protocol from Nanopore (version ENR_9084_v109_rev_04Dec2018). Briefly, 0.75μL of each of the cRNAs (100μM in TE, pH 7.5, IDT) were pooled together. 2μL of this mixture was added to 2μL of tracrRNA (100μM, IDT) and 6μL of Duplex Buffer (IDT). This was mixed and heated at 95°C for 5 min in a Thermal Cycler (Applied Biosystems ProFlex PCR System). 10μL of the annealed cRNA-tracrRNA pool was added to 10μL of NEB CutSmart buffer, 79.2μL of nuclease-free water and 0.8μL of Cas9 nuclease (62μM, IDT Cat. No. 1081059). The ribonucleoprotein complexes (RNPs) were allowed to form by incubating the tube at room temperature for 30 min. The genomic DNA (extracted as described above) was dephosphorylated by adding 1.5μL of rSAP (NEB, Cat. No. M0371S) to 25.5μL of HMW DNA and 3μL of NEB CutSmart Buffer. The tube was mixed and incubated at 37°C for 1 h followed by inactivation of rSAP at 65°C for 10 min. 10μL of the Cas9 RNPs were added to the dephosphorylated DNA along with 1μL of freshly prepared 10mM dATP (NEB, Cat. No. N0440S) and 1μL of Taq polymerase (NEB, Cat. No. M0273). This was incubated at 37°C for 1 h followed by 72°C for 5 min in a Thermal Cycler to cleave the DNA by Cas9 and dA-tail the target DNA. This was followed by adapter ligation, clean-up and flowcell loading following the protocol for SQK-LSK112 sequencing kit (Version GDE_9141_v112_revC_01Dec2021) with the following modifications: Incubation with and elution from AMPure XP beads (Beckman

Coulter, Cat. No. A63882) was extended to 30 min each. Incubation for ligating adapters was extended to 2 h at room temperature. Sequencing reads were basecalled using Guppy (version: 6.0.1 + 652ffd1) using the following config file for high accuracy basecalling: dna_r10.4_e8.1_hac.cfg. Coverage for HYP1 and HYP3 loci was plotted using GenomicRanges and GenomicAlignment in R, adapting the script from Giltpatrick et al.[41]

Motif analysis for HYP1 and HYP3 genes from nanopore and PacBio HiFi sequencing

A custom pattern-matching script was developed to retrieve HVD sequences from Cas9-enriched and PacBio HiFi using the Biostrings and stringr packages in R. All the scripts and notes on how to use them can be found at https://github.com/unnatisonawala/HYPervariable_HYPs - https://zenodo.org/doi/10.5281/zenodo.11108598.

In case of Cas9-enriched Nanopore sequencing from a population of J2s, the reads were filtered additionally filtered for Q > 17 for *G. pallida* and Q > 15 for *G. rostochiensis* using NanoFilt[42] and manually curated for motifs not identified by the pattern-matching script due to Nanopore errors. Species richness for HVD variants were calculated using estimateR function from the vegan[43] package in R.

Simulating HYP HVDs *in silico*

All nanopore-derived unique HYP HVDs (55) were lined up by their start position, and the probability of each motif occurring at each position was computed (including no motif, i.e., the end of an HVD). A custom python script.

(https://github.com/sebastianevda/HYP/tree/main/Simulate_HVDs/Compute_HVDs_based_on_positional_probability_and_known_pairs.py - https://doi.org/10.5281/zenodo.11235347) was written to generate HVDs motif by motif, based on the probability of a given motif at that position (probability of end of HVD at position 29 is 1), provided that the selected motif is known to occur after whatever motif was in current position-1. In so doing, the script will generate many HYP1 HVDs. Finally, this list is triaged to report only those that have >20 motifs, and have known start or end patterns because they seem to be the most conserved arrangements, using a second custom python script (Triage_simulated_HVDs_based_on_known_starts_and_ends.py). One million iterations were performed.

QUANTIFICATION AND STATISTICAL ANALYSIS

Zygosity

To empirically derive the probability of 38/68 individuals being homozygous in spite of the large number of apparently available alleles in the population, a custom python script was written (https://github.com/sebastianevda/HYP/tree/main/Zygosity - https://doi.org/10.5281/zenodo.11235347). The script, based on a population of alleles, randomly selects 2 "parents" for each of 68 individuals, and computes zygosity. The population of alleles used was HVD domains of all 133 HYP1 nanopore reads (i.e., maintaining the frequency of each unique allele). Ten million iterations were performed. After 10 million simulations of selecting two parental alleles from the population for each of 68 nematodes, the highest number of homozygous individuals was 8/68, which occurred once. The probability of 38/68 individuals being homozygous was therefore determined to be substantially less than one in 10 million. No methods were used to determine whether the data met assumptions of the statistical approach.

ADDITIONAL RESOURCES

No external sites have been generated to support discussion or use of the information/data/material created by the manuscript.

Cell Genomics, Volume *4*

Supplemental information

A gene with a thousand alleles:

The hyper-variable effectors

# of plant-parasitic nematodes

Unnati Sonawala, Helen Beasley, Peter Thorpe, Kyriakos Varypatakis, Beatrice Senatori, John T. Jones, Lida Derevnina, and Sebastian Eves-van den Akker

Supplemental Information

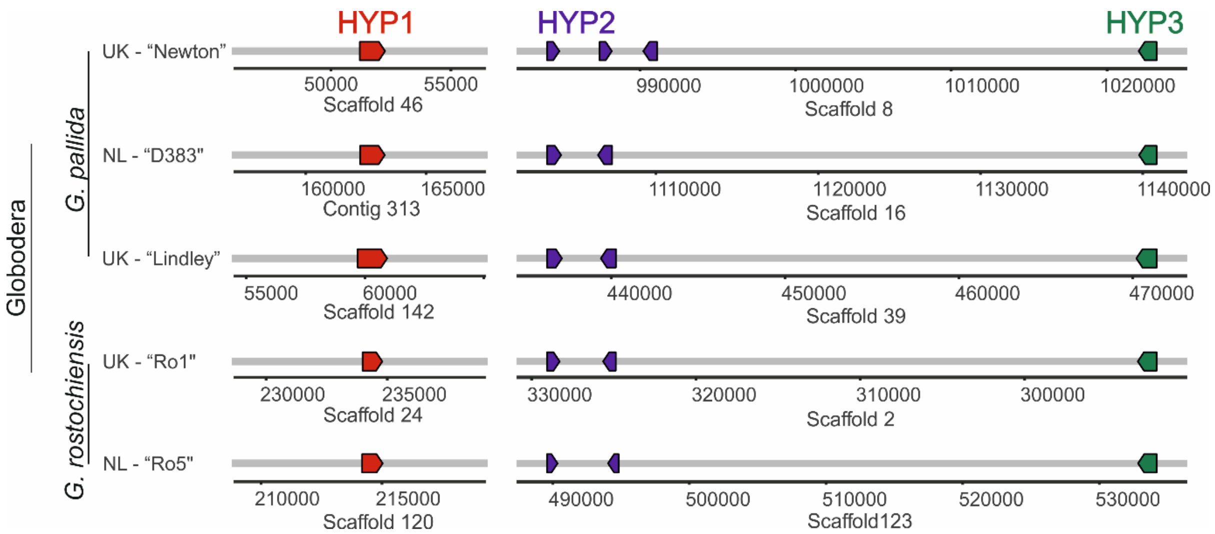

Figure S1. Schematic gene maps indicating positions of HYP1, HYP2 and HYP3 loci across multiple populations/pathotypes of *G. pallida* and *G. rostochiensis,* related to Figure 1.

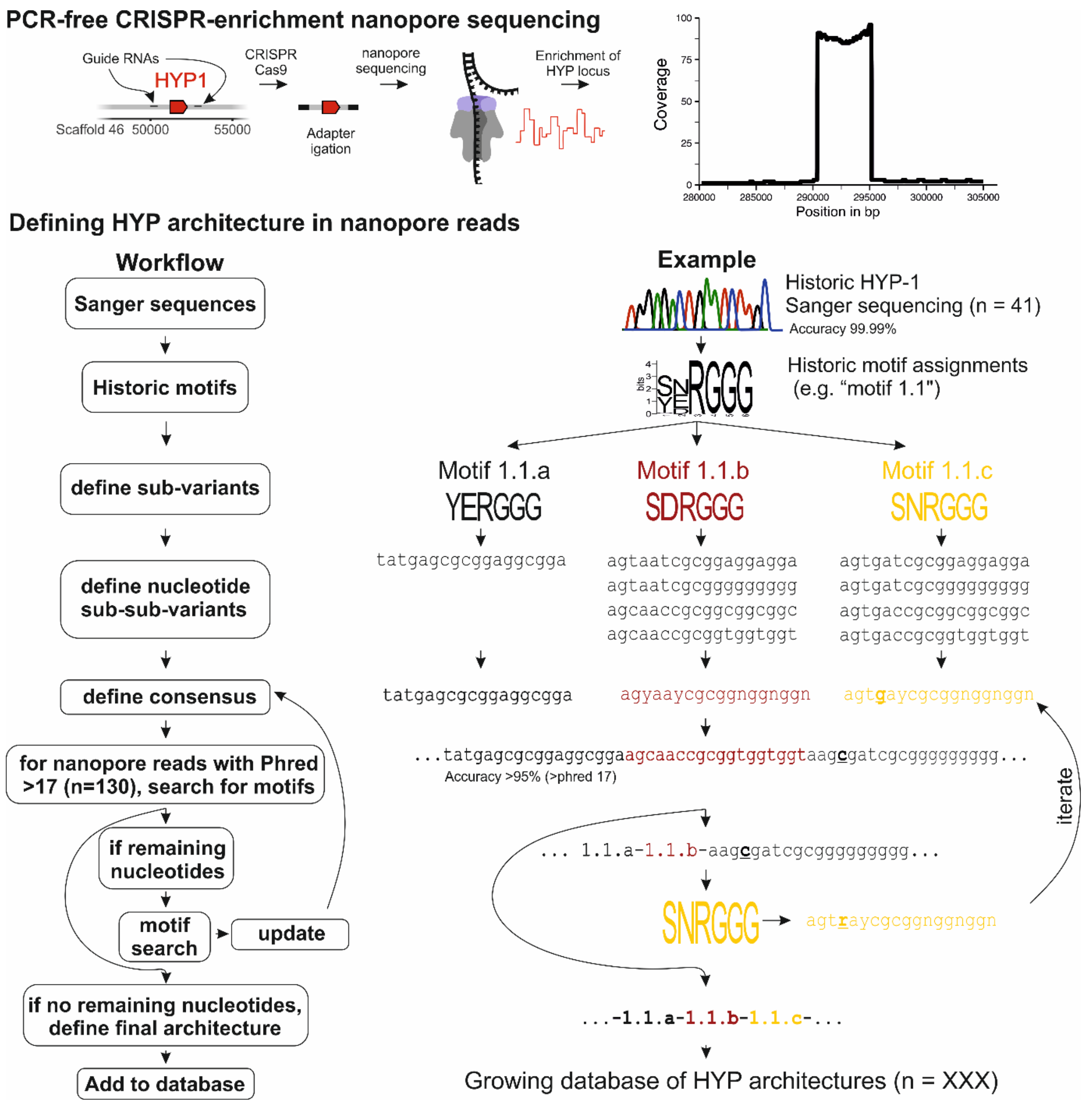

Figure S2. Schematic of workflow for characterization of HYP variants within a population or single nematode, related to Figure 2. A customised workflow was designed by taking advantage of the 'modular' nature of the motifs within the variable region of HYP genes. Previously identified motifs or those identified by MEME were used to build a pattern matching code using the Biostrings package in R. The pattern matching was optimised for error-prone nature of long reads. Several iterations were performed to update code for missed motifs. Finally, known motifs missed due to multiple sequencing errors were manually curated to generate a final list of HYP 'variants' consisting of an uninterrupted string of identified motifs.

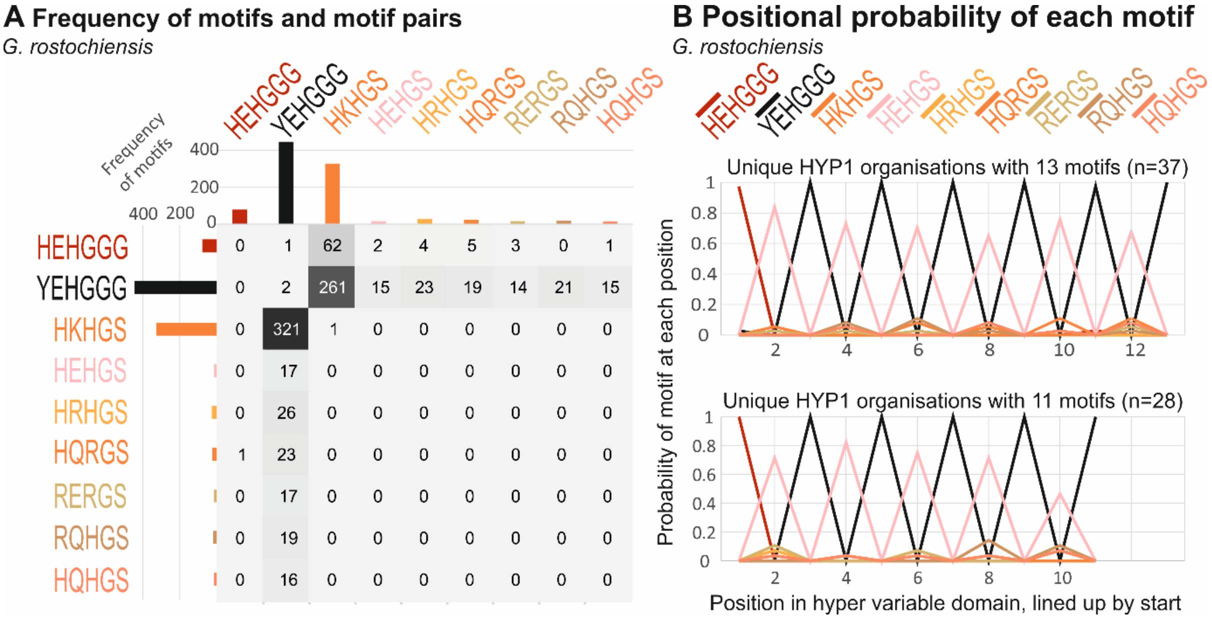

Figure S3. There are different rules underlying *G. rostochiensis* HYP1 variation, related to Figure 2. A) Frequency of motif pairs within HYP1 HVDs. Each motif is shown with amino acid sequence, y axis of matrix is position n and x axis is position n+1. For examples: i) HEHGGG is almost always first (and so almost never follows another motif), ii) all motifs can appear before YEHGGG, but YEHGGG is the only motif that can appear before RQHGS). B) The positional probability of each motif at each position for HYP1s with HVDs containing 13 (top) and 11 (bottom) motifs show an alternating pattern of YEHGGG followed by an other motif.

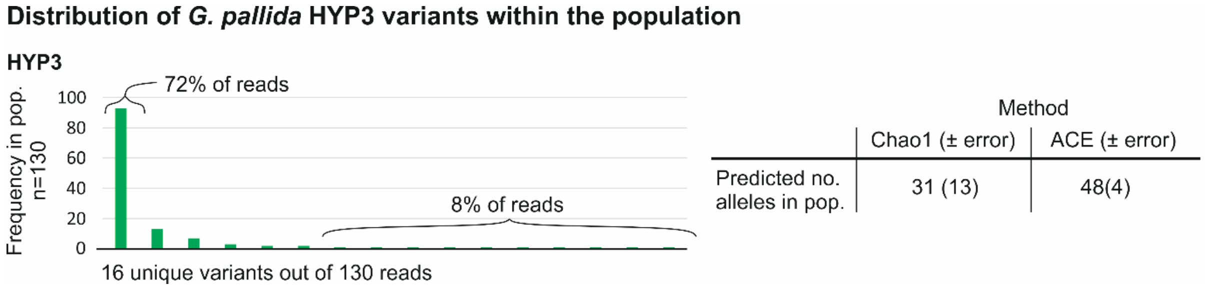

Figure S4. Frequency distribution and population size estimates of *G. pallida* HYP3 alleles, related to Figure 3. The observed occurrences of each unique HYP variant when sampling a population (n= 125). Inset are two independent species abundance estimates for the total number of alleles in the population, based on the sampling.

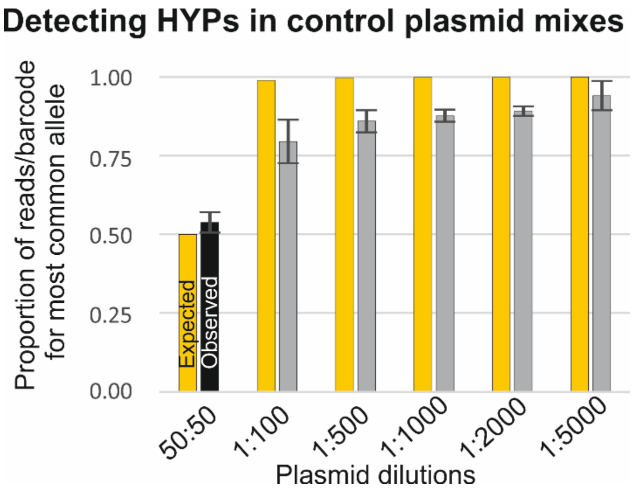

Figure S5. Detecting HYPs in control plasmid mixes, related to Figure 4. Comparison of observed (black and grey) and expected (yellow) proportions of reads per barcode pair that are contributed by the most common allele, for a range of known plasmid dilutions.

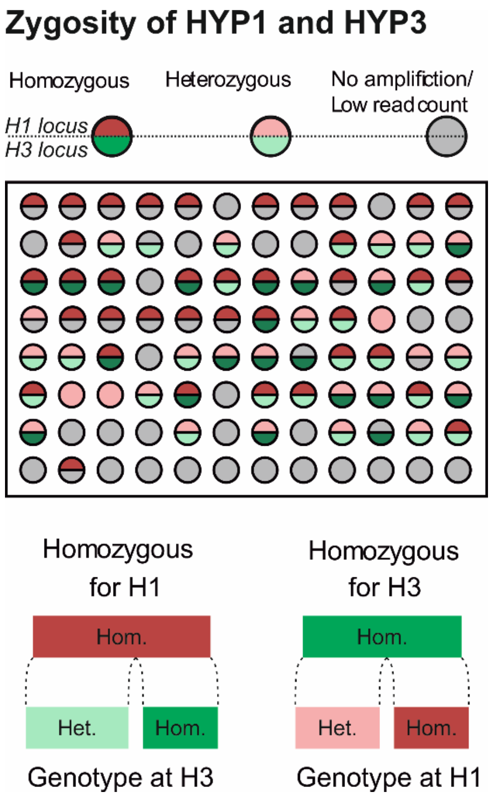

Figure S6. Zygosity of HYP1 and HYP3 loci for 68 J2s, related to Figure 4. Zygosity of HYP1 locus (reds) is unlinked to zygosity of HY3 locus (greens).

Metrics	*G. pallida* 'Lindley'[5] shortread assembly	*G. pallida* 'Newton' longread assembly	*G. pallida* 'Lindley' longread assembly	*G. rostochiensis* 'Ro1'[2] shortread assembly	*G. rostochiensis* 'Ro1' longread assembly
**Size (Mb)**	124.6	119.6	109.1	95.9	102.2
**Scaffolds(n)**	6,873	173	672	4281	267
**Scaffold N50 (bp)**	121,687	2,225,126	595,505	88,688	2,199,277
**Longest scaffold (bp)**	600,076	8,303,752	4,690,510	688,384	8,068,213
**GC (%)**	37	37	37	38	39
**Ns (bp)**	21,024,229	1,237,729	2,170,753	4,399,212	1,328,123
**BUSCO (%)**	Complete:73% [Duplicated:13%], Fragmented:12%, Missing:14%,	Complete: 89% [Duplicated: 9.9%],Fragmented: 5.9%, Missing: 4.6%	Complete: 86% [Duplicated: 12%], Fragmented: 5.2%, Missing: 8.2%	Complete: 88% [Duplicated: 9.5%], Fragmented: 5.9%, Missing: 5.9%	Completed:87% [Duplicated:9.2%],Fragmented :5.9%,Missing:6.6%
**Predicted genes (n)**	16,466	16,321	22,426	14,378	14309

Table S1: Genome metrics for Globodera genome assemblies, related to Figure 1.

Table S2. Table of crRNAs used for targeting HYP1 and HYP3, related to Figure 1.

Target	Forward	Reverse
HYP1 (*G.pallida*)	ATTGTATGATTGTCAAAATC<u>CGG</u>	AATCAAATGACACATTACAA<u>TGG</u>
HYP3 (*G.pallida*)	TTATGTCGATGCGTCCGAGT<u>TGG</u>	CGGATCCAACCCCCGGAACG<u>CGG</u>
HYP1 (*G.rostochiensis*)	AATAACTCCCGGCGACGTCG<u>CGG</u>	TCTCTAGTCCGAACGTCGAA<u>AGG</u>
HYP3 (*G.rostochiensis*)	CAGCCCAATTGCAGGATGTT<u>TGG</u>	CCGATTCTACGTCTTATGAT<u>AGG</u>
